# Matrix stiffness drives alterations in aldehyde metabolism, inducing DNA damage and transformation

**DOI:** 10.1038/s41598-025-12880-0

**Published:** 2025-08-09

**Authors:** Matthew Jones, Hannah Percival, Alis Hales, Amber Wood, Heyuan Sun, Fabianna Tennant, Eleanor Broadberry, Eldhose Skaria, Harry Barnes, Egor Zindy, Craig Lawless, Charles Streuli, Joe Swift, Keith Brennan, Andrew P. Gilmore

**Affiliations:** 1 Manchester Cell Matrix Centre, Faculty of Biology, Medicine and Health, University of Manchester, Oxford Road, Manchester, M13 9PT UK; 2 Division of Cancer Sciences, Faculty of Biology, Medicine and Health, University of Manchester, Oxford Road, Manchester, M13 9PT UK; 3 Division of Cell Matrix Biology and Regenerative Medicine, Faculty of Biology, Medicine and Health, University of Manchester, Manchester, UK; 4 Center for Microscopy and Molecular Imaging, Université libre de Bruxelles, B-6041 Gosselies, Belgium

**Keywords:** Cancer, Breast cancer, Cancer metabolism, Cancer models, Cell biology, Cell adhesion

## Abstract

Microenvironmental stiffness regulates fundamental aspects of cell behaviour, including proliferation, differentiation and metabolism, many of which are implicated in cancer initiation and progression. In the mammary gland, extracellular matrix (ECM) stiffness, associated with high mammographic density, is linked to increased breast cancer incidence. However, a mechanistic link between increased ECM stiffness and the genomic damage required for transforming mutations remains unclear. Here we show that ECM stiffness induces changes in mammary epithelial cell (MEC) metabolism which drive DNA damage. Using a mechanically tunable 3D-culture model, we demonstrate that transcriptional changes in response to increased ECM stiffness impair the ability of MECs to remove reactive aldehydes. Downregulation of multiple aldehyde dehydrogenase isoforms in MECs within a stiffer 3D ECM leads to higher levels of reactive aldehydes, resulting in genomic damage and transformation. Together, these results provide a mechanistic link between increased ECM stiffness and the genomic damage required for breast cancer initiation.

**Supplementary Information:**

The online version contains supplementary material available at 10.1038/s41598-025-12880-0.

## Introduction

Cells are exposed to a wide range of mechanical stimuli within their microenvironment, such as stiffness of the extracellular matrix (ECM) or cyclical strain as in lung or cardiac muscle^1,2^. Mechanotransduction is the conversion of such mechanical stimuli into biochemical signals regulating gene expression and behaviour. Different tissues have distinct mechanical properties which cells interpret through integrin- and cadherin-based adhesion complexes^3,4^. Signalling pathways coupled to these adhesion complexes then elicit mechanosensitive intracellular responses including differentiation^5^, proliferation^6^ and apoptosis^7^.

Variations in the normal range of tissue mechanics are associated with diseases such as fibrosis and cancer. It is well established that increased ECM stiffness can drive cancer progression and invasion through altered mechanotransduction^8,9^. However, microenvironmental stiffness is also implicated in cancer initiation. Mammographic density (MD) is defined as the area of radio-opaque fibroglandular tissue seen on a mammogram. High MD represents the second largest independent risk factor for breast cancer and is associated with increased stiffness within the periductal stroma of the breast^10–12^. The resulting changes in mechanostransduction promote altered breast cell behaviour^12^. However, the mechanisms by which this might promote increased genomic damage, necessary for acquiring oncogenic mutations, remain unclear.

Here we asked how changes in mechanotransduction within mammary epithelial cells (MECs) might drive genomic damage. We used a 3D hydrogel system which can be mechanically tuned to mimic variations seen in the tissue microenvironment^13^. MEC acini within a stiff microenvironment developed a pre-malignant phenotype characterised by excessive and irregular growth, a loss of tissue specific gene expression, and increased acquisition of anchorage-independence. Global comparison of gene and protein expression between MECs grown in soft or stiff 3D ECM highlighted broad changes in metabolic pathways, including decreased expression of several isoforms of aldehyde dehydrogenase (ALDH) when MECs were cultured in stiff ECM. Downregulation of ALDH resulted in increased accumulation of reactive aldehydes and DNA damage. These changes were mediated through RhoA-dependent mechanosignalling. Together these results establish a link between ECM mechanotransduction and genomic damage through altered metabolism of reactive aldehydes.

## Results

### Increased 3D ECM stiffness inhibits mammary epithelial cell differentiation

**Fig. 1:**
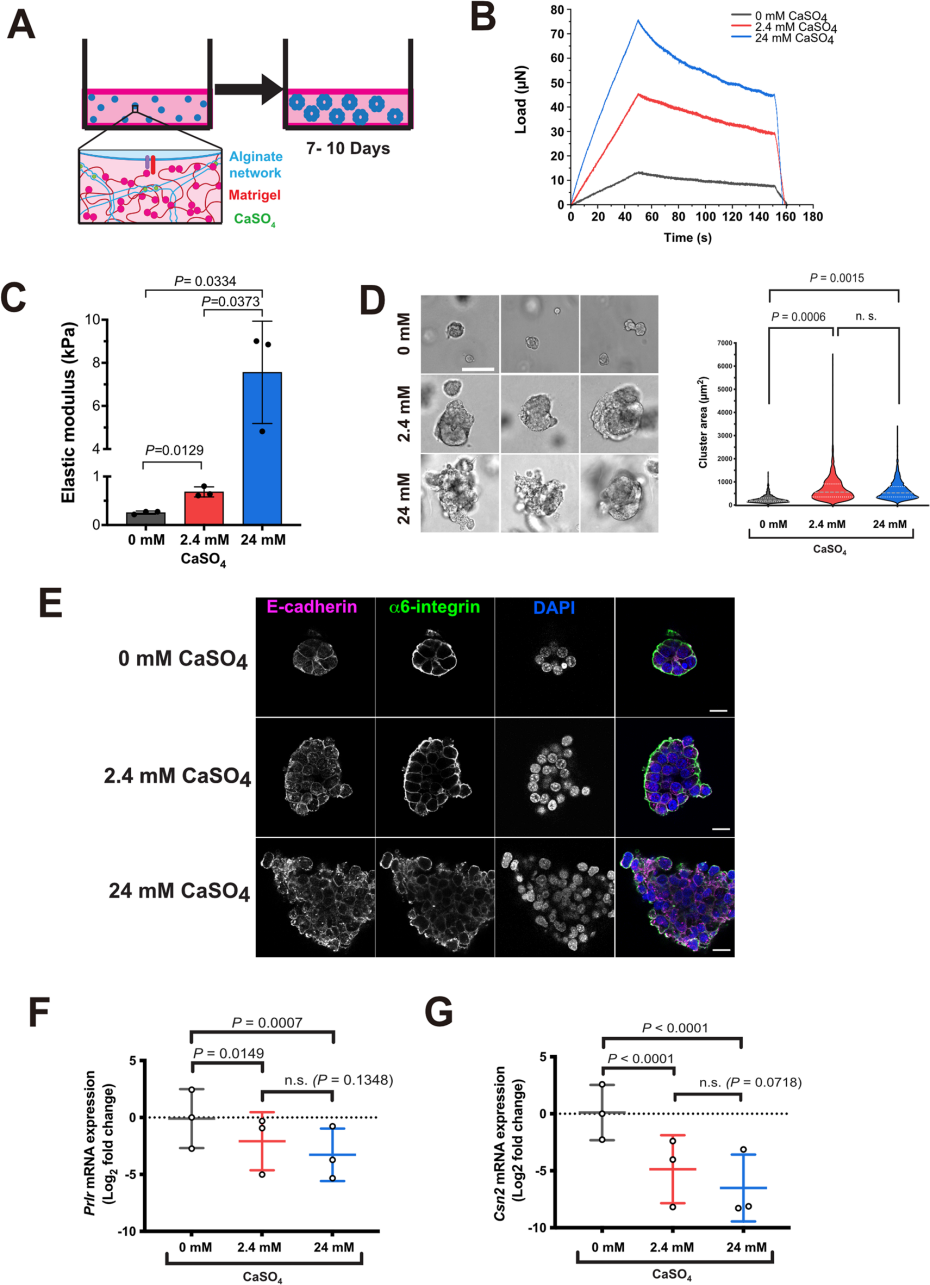
Increased ECM stiffness inhibits differentiation of MECs. **A.** Schematic diagram of the interpenetrating network of MAG hydrogels. **B.** Representative stress-relaxation curves for MAG hydrogels with either no CaSO_4_ or at 2.4 mM or 24 mM. **C**. Elastic modulus calculated from the data in B. for three independent replicate hydrogels at each CaSO_4_ concentration. Each hydrogel was measured in three separate locations, and the individual data points represent the mean of these three measurements. Data shown as mean ± SD for the three replicate gels. **D**. Representative brightfield images of EpH4 acini cultured in MAG hydrogels with 0 mM, 2.4 mM and 24 mM CaSO_4_ for 10 days. Right panel shows quantification of the area of acinar clusters formed after 10 days. At least 600 acini were measured per condition. Scale bars, 50 mm. Significance was determined by one-way ANOVA (n=3). **E**. Immunofluorescence (IF) images of EpH4 acini cultured in Matrigel-Alginate hydrogels with 0 mM, 2.4 mM and 20 mM CaSO_4_ for 10 days. Acini were extracted from hydrogels and immunostained for E-cadherin and α6-integrin. Nuclei were stained with DAPI. Scale bars, 30mm. **F.** Log_2_ fold-change in gene expression of *Prlr*, normalised to *Gapdh*, as determined by RT-qPCR in EpH4 cells. Mean ± SD, *n* = 3 per condition, across independent experiments (represented by data points). Two-way ANOVA with Tukey’s post-hoc test. **G. **Log_2_ fold-change in gene expression of *Csn2*, normalised to *Gapdh*, as determined by RT-qPCR in EpH4 cells. Mean ± SD, *n* = 3 per condition, across independent experiments (represented by data points). Two-way ANOVA with Tukey’s post-hoc test.

Both normal and transformed MECs undergo phenotypic changes in response to the mechanical stiffness of their microenvironment^9,13,14^. To understand how changes in mechanosignalling might promote early events in MEC transformation, we utilised a previously described 3D-culture model composed of interpenetrating networks of Matrigel and alginate^13^. These gels are mechanically tuneable via crosslinking with divalent cations (Fig. 1A-C). We wanted to ensure that the range of stiffness of gels used represented that of low and high MD breast tissue.

Measurements of breast tissue stiffness vary, although a recent study reported mean values for elastic modulus in the range of 1 kPa to 2 kPa for low and high MD tissue respectively, measured by atomic force microscopy^12^. However, that study showed considerable variation in mean elastic modulus values between individuals, ranging from below 1 kPa to near 6 kPa. Addition of calcium sulphate (CaSO_4_) up to 20 mM has been reported to generate Matrigel/Alginate (MAG) gels with storage moduli ranging from 3 Pa to 200 Pa, based on oscillatory shear rheometry^13^. One issue with Matrigel is the inherent batch variation leading to variability in mechanical properties^15,16^. We therefore undertook mechanical characterisation of our MAG hydrogel preparations by load-relaxation testing, using gels with 0 mM, 2.4 mM and 24 mM CaSO_4_ (Fig. 1B). MAGs were determined to be ~ 200 Pa, ~ 700 Pa and ~ 7.5 kPa for 0 mM, 2.4 mM and 24 mM CaSO_4_ respectively (Fig. 1C).

To determine how MAG stiffness altered MEC function, we utilised EpH4 cells, a non-transformed murine luminal epithelial cell line^17^. Like primary MECs, EpH4 cells form polarised acini within 3D Matrigel that express milk proteins, and thus provide a functional model of MEC differentiation^18^. Single cell suspensions of EpH4 cells were embedded within 3D MAG hydrogels, either without additional Ca^2+^_,_ or with CaSO_4_ at 2.4 mM or 24 mM (Fig. 1D). After 10 days EpH4 acini were significantly larger within MAG hydrogels incorporating either 2.4 mM or 24 mM CaSO_4_, compared to those with 0 mM CaSO_4_. To assess their organisation and polarity, intact EpH4 acini were extracted from MAG hydrogels after 10 days and immunostained for E-cadherin and α6-integrin (Fig. 1E). In soft gels (0 mM CaSO_4_) EpH4 acini showed clear basal and lateral organisation of α6-integrin and E-cadherin respectively. In 2.4 mM CaSO_4_ hydrogels, α6-integrin was still predominantly basal, but acini appeared less organised. In 24 mM CaSO_4_ there was little discernible polarity seen through integrin localisation. Thus, ECM stiffness progressively altered EpH4 acinar organisation.

To examine MEC differentiation, we performed quantitative reverse transcriptase PCR (RT-qPCR) to measure relative expression of prolactin receptor (*Prlr*) and beta-casein (*Csn2*) (Fig. 1F, G). MEC acini in hydrogels with 2.4 mM or 24mM CaSO_4_ showed significantly reduced expression of *Prlr* and *Csn2* compared to those in soft MAG. There was no significant difference in *Prlr* and *Csn2* expression between cells grown in either 2.4mM or 24 mM CaSO_4_. Although the Ca^2+^ crosslinking the hydrogel should be sequestered by the alginate^13^, to confirm that changes in gene expression were not due to differences in free Ca^2+^ we analysed gene expression of parathyroid hormone-related protein (PTHrP, *Pthlh*). *Pthlh* expression is supressed by calcium in non-transformed MECs^19^. There was no suppression of *Pthlh* expression when EpH4 cells were grown in MAG with or without 2.4 mM or 24 mM CaSO_4_ (Fig.S1A). 2.4 mM CaSO_4_ hydrogels had similar effects on EpH4 gene expression as the 24 mM condition. However, the 24 mM hydrogels showed greater gel to gel variation in storage modulus (Fig. 1C). Furthermore, acini within the 2.4 mM CaSO_4_ MAG hydrogels still showed basal polarisation of α6 integrin (Fig. 1E). We therefore hypothesised that this would be more reflective of variations in periductal stiffness in vivo, and all subsequent experiments used 2.4 mM CaSO_4_ as “stiff” and no additional Ca^2+^ as “soft”.

Breast cancer is often associated with reversion of MECs to a more stem-like state. We therefore asked whether ECM stiffness induced changes in lineage-specific marker expression. We used RT-qPCR to compare marker gene expression in EpH4 cells grown in soft and stiff MAG (Fig.S1B). There were no significant changes in the expression of markers of mammary stem cell (*Sox9*, *Egr2*, or *Snai2*), myoepithelia (*Krt14* and *Vim*) or differentiated luminal epithelia (*Krt18* and *Cdh1*). Although the luminal progenitor marker *Elf5* did show significantly reduced expression in stiff conditions relative to soft, these data overall did not indicate a significant change overall.

Together, these results suggest that increased 3D ECM stiffness disrupts the differentiation and organisation of MECs.

### Increased ECM stiffness drives proliferation and DNA damage in MECs

**Fig. 2:**
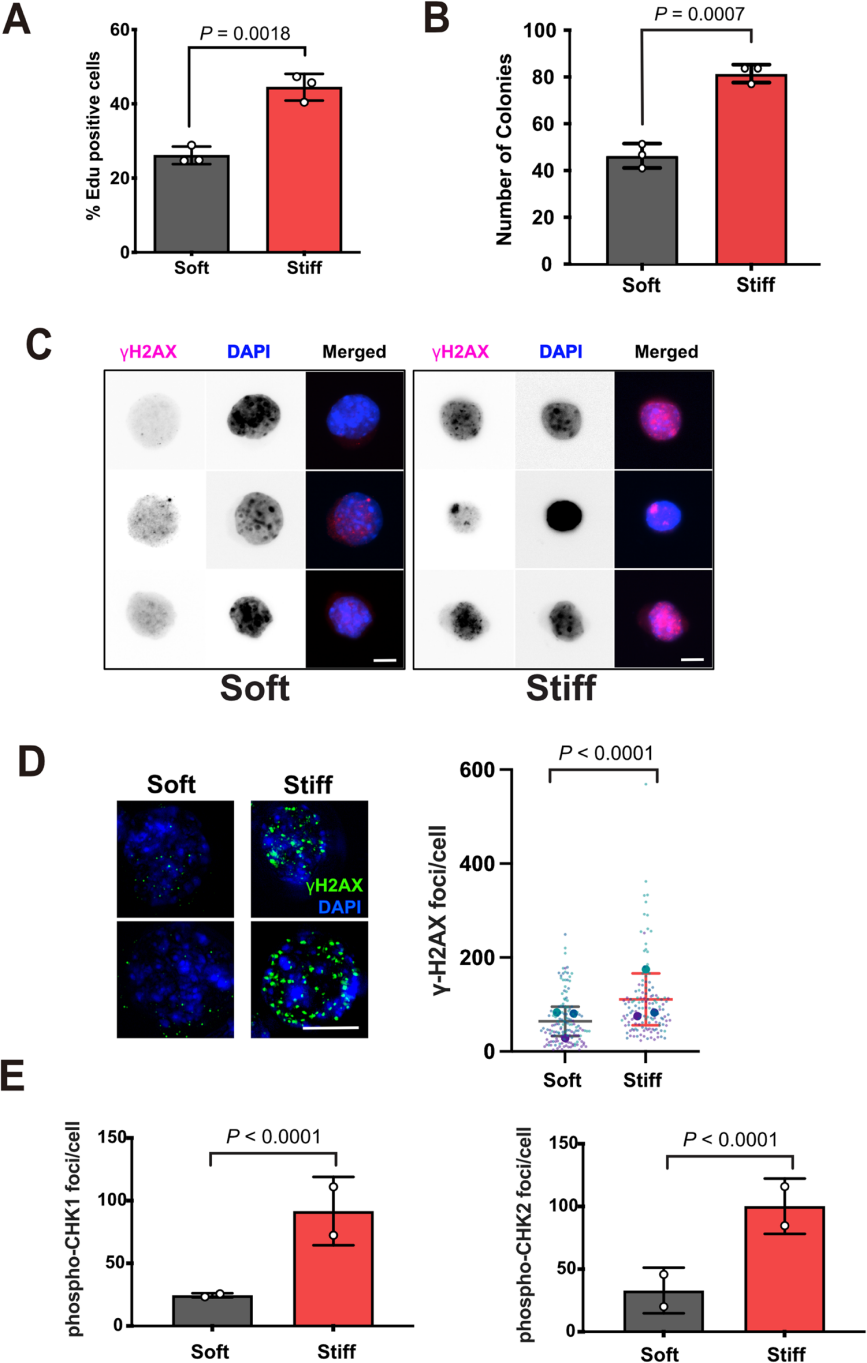
Increased ECM stiffness induces DNA damage in MECs **A.** EpH4 cells were grown in soft or stiff MAG hydrogels for 10 days and labelled with EdU for the last 5 hours prior to isolation and fixation. The percentage of EdU-positive cells were quantified. Mean ± SD from three independent experiments. Two-tailed Student’s *t*-test. **B**. Number of colonies formed in soft agar following culture in soft or stiff 3D ECM for seven days. Colonies were quantified after 21 days in soft agar. Mean ± SD, *n* = 3 per condition from independent experiments, each performed in triplicate. Data points represent mean number of colonies from triplicates for each independent experiment. Two-tailed Student’s *t*-test. **C**. Representative IF images of EpH4 cells immunostained for phospho-γH2AX (Ser139) following culture in 3D MAG gels of different stiffnesses for 24 hrs (scale bars, 10 mm). **D.** Quantification of number of γH2AX (phospho-Ser139) foci/cell. Left panel shows representative deconvolved IF images of nuclei immunostained for phospho-γH2AX and foci quantified. Scale bar = 10 µm. Mean ± SD, *n* = 3 per condition from independent experiments. Small data points are foci from individual nuclei in each independent experiment, large data points represent mean number of foci for each independent experiment, calculated from 40-50 cells/condition. Two-way ANOVA. **E. **Quantification of phospho-Chk1 (Ser345) and phospho-Chk2 (Thr68) in EpH4 cells cultured in soft or stiff 3D ECM for 24 hrs. Mean ± SD, *n* = 2 per condition from independent experiments, each performed in duplicate. Data points represent the mean number of foci for each independent experiment, calculated from 20-30 cells/condition.

As acini grown in stiff MAG were larger than those grown in soft, we compared proliferation between the two conditions. EpH4 cells were embedded into soft and stiff MAG hydrogels for 10 days and labelled with EdU for 5 h prior to fixation. EdU- positive cells were then quantified (Fig. 2A). Significantly more cells incorporated EdU in the stiff MAG compared to soft, indicating stiffness increased proliferation. We then asked if culturing MECs in a stiffer 3D ECM led to anchorage independent growth in a soft agar colony formation assay^20^. EpH4 acini cultured in soft or stiff 3D MAG gels for 7 days were extracted, dissociated into single cells, and re-seeded into soft agar for a further 21 days. Cells isolated from acini passaged through the stiff MAG subsequently formed more anchorage independent colonies in soft agar than those from soft hydrogels (Fig. 2B).

We hypothesised that the increased number of anchorage-independent colonies formed by EpH4 cells grown in stiff MAG may be due to greater DNA damage accumulation in these cells compared to those from soft MAG. To assess DNA damage, we visualised Ser139 phosphorylation of histone H2AX (γH2AX) to visualise double-stranded DNA breaks^21^. EpH4 cells were seeded into soft or stiff MAG hydrogels, acini isolated, dissociated into single cells and immunostained for γH2AX (Fig. 2C). Cells isolated from stiff gels showed more γH2AX staining than those from soft gels. Quantification of the number of γ-H2AX foci per nucleus indicated that there were significantly more γH2AX foci when cells had been cultured within stiff ECM compared to soft, indicating that they had acquired more DNA damage (Fig. 2D). We confirmed this by immunostaining cells with antibodies to phosphorylated Chk1 (Ser345) and Chk2 (Thr68), downstream markers of ATM and ATR activation (Fig. 2E). Both Chk1 and Chk2 showed increased phosphorylation in cells isolated from the stiff MAG compared with soft.

Taken together, these results show that MECs accumulate greater levels of DNA damage leading to transformation when cultured in a stiffer 3D ECM.

### ECM stiffness alters global gene expression and downregulates Aldh isozymes, resulting in increased accumulation of reactive aldehyde species

**Fig. 3:**
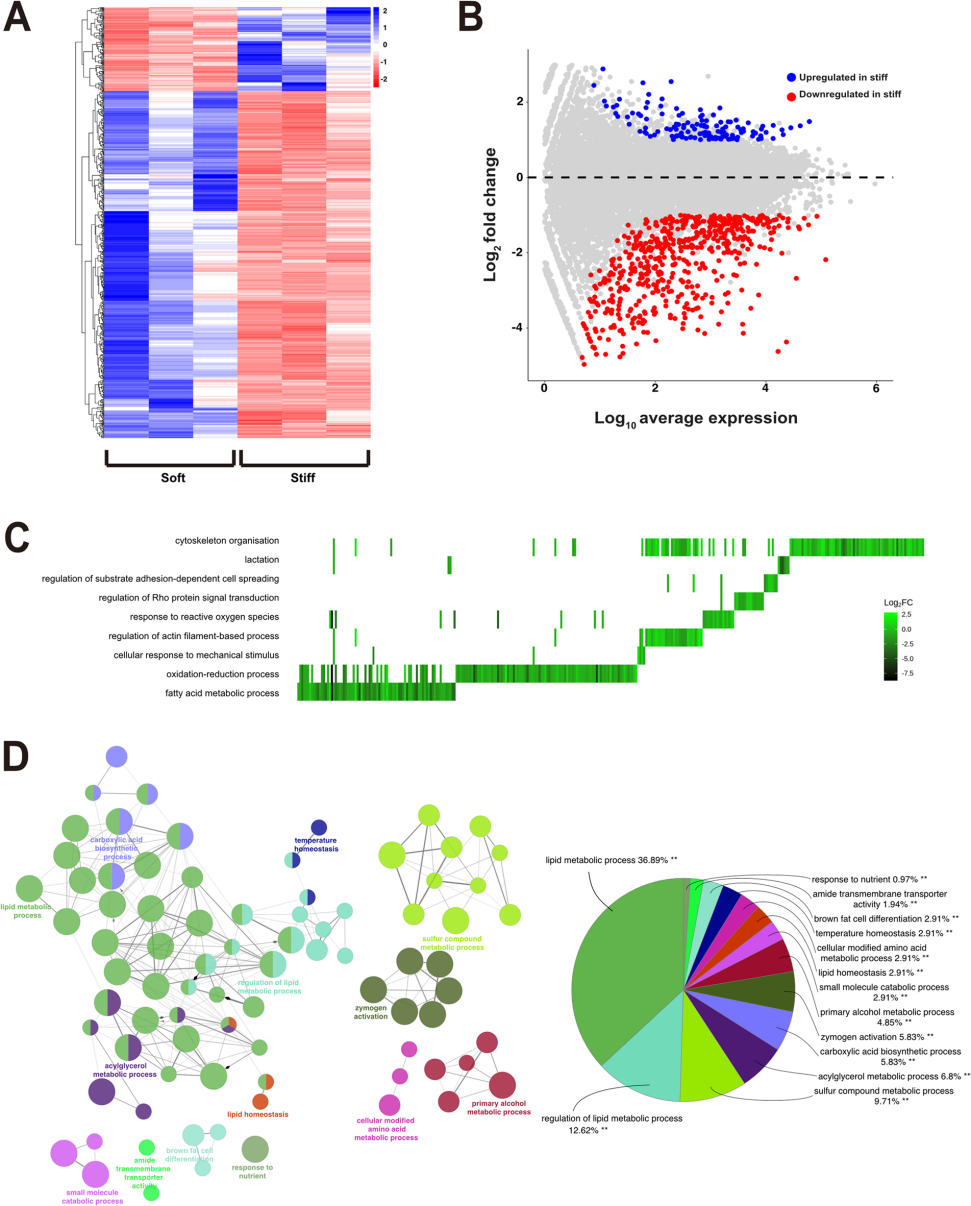
Increased ECM stiffness alters global gene expression in MECs**. A.** Hierarchical clustering heatmap representing significantly differentially expressed genes in EpH4 cells cultured in 3D gels of different stiffnesses, as determined by RNAseq. Three independent biological replicates of both soft and stiff 3D cultures are shown. **B.** MA plot generated from RNAseq data, showing genes that are significantly upregulated (blue) and downregulated (red) in EpH4 cells grown in the stiff condition, relative to soft. Data shown are the mean from the 3 biological replicates in A. **C.** Heatplot showing significant GO terms associated with differentially expressed genes, and their Log_2_ fold-change values in the stiff condition, relative to soft. **D**. GO analysis of significantly downregulated genes in stiff relative to soft conditions (adjusted p< 0.05 and absolute fold change ≥1.5) presented as a functionally grouped network. Significantly enriched biological process GO terms (adjusted p< 0.05) are represented as nodes and clustered into functional groups based on their overlapping genes (kappa score ≥ 0.4). Nodes are colour-coded by functional group and node size represents the significance of GO term enrichment. To reduce network complexity, only the most significant GO term within each functional group is annotated on the network. Pie chart showing the percentage of total genes assigned to each functional group in the GO analysis. generated using the ClueGO plugin within Cytoscape (v.2.5.10).

To determine how increased ECM stiffness might drive DNA damage and MEC transformation, we performed an unbiased global transcriptome analysis using RNA sequencing (RNAseq). EpH4 acini were grown from single cells in either soft or stiff MAG, mRNA isolated from three independent replicates and gene expression across the transcriptome quantified (Fig. 3A). Overall, approximately 1500 genes showed differential expression between EpH4 cells cultured in soft and stiff 3D ECM (Fig. 3B). Most differentially expressed genes showed reduced expression in stiff relative to soft MAG. These mechanosensitive differences in EpH4 gene expression were specific to cells grown within 3D matrices, as EpH4 cells grown on 2D soft and stiff hydrogel substrates showed few significant differences in gene expression (Fig.S2A, B). Interestingly, comparing the gene expression profiles across both experiments indicated that the most significant differences were between EpH4 cells grown in 2D vs. 3D. Indeed, MECs grown on 2D hydrogels did not express key markers of differentiation, including *Csn2* and *Prlr *(Fig.S2C). To validate the RNAseq data, we specifically analysed expression of genes associated with MEC differentiation assessed earlier using RT-qPCR. In agreement with the RT-qPCR, these genes, including *Csn2* and *Prlr*, were significantly downregulated in EpH4 acini grown within stiff compared to soft 3D hydrogels (cf. Fig.S2C and Fig. 1F, G). There were no significant changes in *Pthlh* expression between soft and stiff 3D, or 2D vs. 3D (Fig.S2C).

We next performed gene ontology (GO) enrichment analysis on the 3D RNAseq data (Fig. 3C). As well as lactation, we observed differential enrichment of terms associated with mechanosensing, including regulation of substrate adhesion-dependent cell spreading, cytoskeleton organisation, actin filament-based process and Rho signalling. These confirmed that EpH4 cells were responding to ECM stiffness through changes in mechanotransduction. Other significant changes were observed with terms relating to metabolic processes. These included fatty acid metabolism, response to reactive oxygen species, and oxidation/reduction processes. As several recent studies have identified that metabolic processes are sensitive to mechanosignalling^22,23^, we speculated that changes in these processes might be linked to increased DNA damage. We specifically asked if there was downregulation in DNA repair pathways. However, whilst not individually significant, there was an overall increase in gene expression in all the major DNA repair pathways in stiff relative to soft MAG (Fig.S3A-E).

**Fig. 4:**
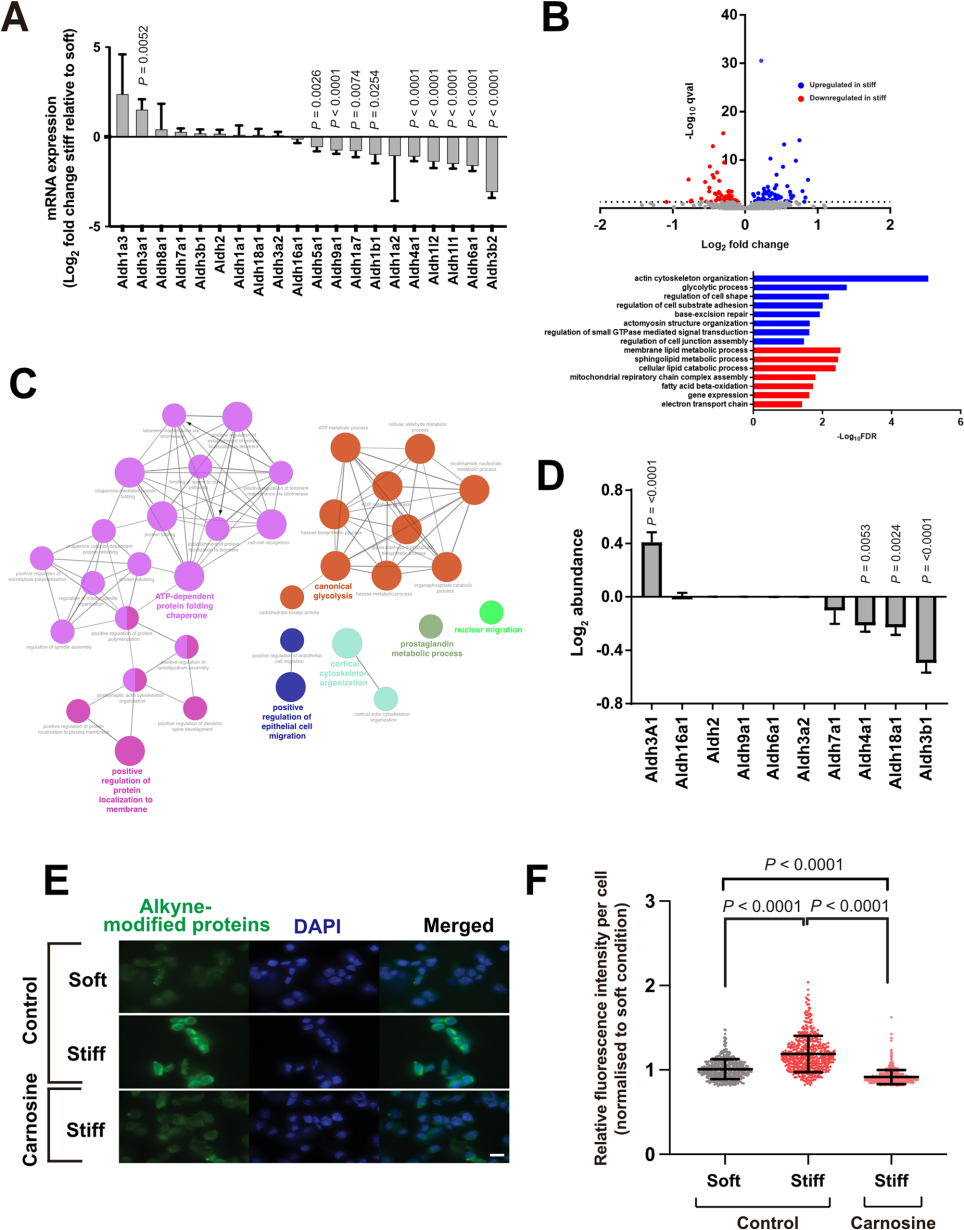
ECM stiffness-induced downregulation of aldehyde dehydrogenases impairs the ability of MECs to oxidise reactive aldehydes. **A.** Log_2_ fold-change in expression of Aldh isozymes in EpH4 cells grown in the stiff 3D ECM relative to soft, as determined by RNAseq. Error bars represent SE (*n* = 3), and statistical significance was determined using DESeq2. **B. **Volcano plot of proteins which were significantly upregulated (blue) and downregulated (red) in cells cultured in stiff conditions compared to soft. Data shown were generated from three independent biological replicates of both soft and stiff 3D cultures. PANTHER GO enrichment analysis of the mass spectrometry results shows GO terms which were significantly enriched due to association with proteins identified in stiff (blue) and soft (red) conditions. **C. **Gene ontology (GO) analysis of significantly upregulated proteins in stiff relative to soft conditions (adjusted p< 0.05) presented as a functionally grouped network. Significantly enriched biological process GO terms (adjusted p< 0.05) are represented as nodes and clustered into functional groups based on their overlapping proteins (kappa score ≥ 0.4). Nodes are colour-coded by functional group and node size represents the significance of GO term enrichment. The most significant GO term within each functional group is annotated in bold on the network. **D. **Log_2_ fold-change in expression of Aldh isozymes in EpH4 cells grown in the stiff 3D ECM relative to soft, as determined by mass spectrometry. Error bars represent SE (*n*= 3), and statistical significance was determined using MSqRob. **E. **Representative fluorescent images of EpH4 cells grown in soft or stiff 3D ECM, with or without addition of carnosine. Cells were isolated from MAG hydrogels and dissociated into single cells, followed by staining with Click-iT™ Lipid Peroxidation Imaging Kit. Scale bar, 20µm. **F.** Single cell quantification of alkyne modified proteins in each condition, based on fluorescent intensity. Mean ± SD, (*n*= 510 cells/condition, across two independent experiments). Data points represent the fluorescence intensity of individual cells, normalised to the median value for the soft, untreated condition. Kruskal-Wallis test with Dunn’s multiple comparisons post-hoc test.

We scrutinised the RNAseq dataset to identify the molecular function terms associated with differentially expressed genes that were downregulated in stiff relative to soft (Fig. 3D, Fig.S4A). Some of the most prominent changes were linked with oxidoreductase activity and fatty acid metabolism, both of which involve oxidation of aldehydes. When we interrogated the list of genes involved, some of the largest differences in expression observed were in genes coding for multiple aldehyde dehydrogenase (Aldh) isoforms. Seven isoforms of Aldh were significantly downregulated at the mRNA level in stiff MAG compared to soft (Fig. 4A). Aldehydes are highly reactive intermediary products of many metabolic pathways and represent a major source of endogenous DNA damage^24^. As such, changes in a cell’s ability to oxidise aldehydes might lead to their accumulation, resulting in DNA damage. Interestingly, *Aldh1a1*, the isoform frequently used as a marker of breast cancer stem cells^25^, was not significantly altered between the soft and stiff MAG (Fig. 4A), in agreement with our observation that there was no shift in mammary epithelial cell lineage **(**Fig.S1B). Similar analysis of genes upregulated in stiff relative to soft revealed there were no significant GO terms enriched.

We performed mass spectrometry to determine whether the changes observed in the RNAseq translated into changes at the protein level. Although the coverage with proteomics is less than that of RNAseq, we identified around 140 differentially expressed proteins between cells cultured in soft and stiff 3D ECM (Fig. 4B). GO analysis suggested that many proteins upregulated in stiff ECM were associated with cellular responses to mechanical stimuli, including actin cytoskeleton organisation, regulation of cell substrate adhesion and small GTPase-mediated signal transduction. Processes altered in stiff MAG were again predominantly associated with lipid metabolism, glycolysis, mitochondrial respiration and electron transport, and aldehyde metabolism (Fig. 4C, Fig.S4B). At the proteomic level, three Aldh isozymes were significantly downregulated in the stiff ECM compared to soft (Fig. 4D). Interestingly, the most marked change was in Aldh3b1, which shares both functional and sequence homology with Aldh3b2, the most downregulated Aldh gene in the RNAseq results (Fig.S2D)^26^.

We asked whether Aldh downregulation resulted in accumulation of reactive aldehydes. To do this, we employed an indirect assay to measure the ability of cells to oxidise aldehydes. We cultured EpH4 cells in soft and stiff MAG for 24 hrs prior to incubation with alkyne-modified linoleic acid (LAA). LAA incorporates into cell membranes, where it is oxidised during lipid peroxidation, a major source of endogenous aldehydes. When LAA is oxidised to hydroperoxides, these subsequently decompose to aldehydes which can modify proteins at nucleophilic side chains, leaving an alkyne group detectable by Click-iT fluorescent staining. Thus, we would expect to see greater levels of alkyne-modified proteins if Aldh activity was suppressed. Following LAA incubation, EpH4 cells cultured in soft or stiff MAG were isolated, dissociated into single cells and stained with Alexa Fluor 488 Azide (Fig. 4E). Fluorescence intensity was quantified for single cells within each population (Fig. 4F). There were significantly higher levels of alkyne-modified proteins in cells isolated from stiff 3D ECM compared to those from soft. The increased protein modification in stiff MAG was abolished by the addition of carnosine, a scavenger of unsaturated aldehydic lipid oxidation products. These results show that increased ECM stiffness results in downregulated expression of *Aldh* genes and impaired oxidation of reactive aldehydes.

### Clearance of reactive aldehydes protects MECs from DNA damage in cells cultured in stiff MAG hydrogels

**Fig. 5:**
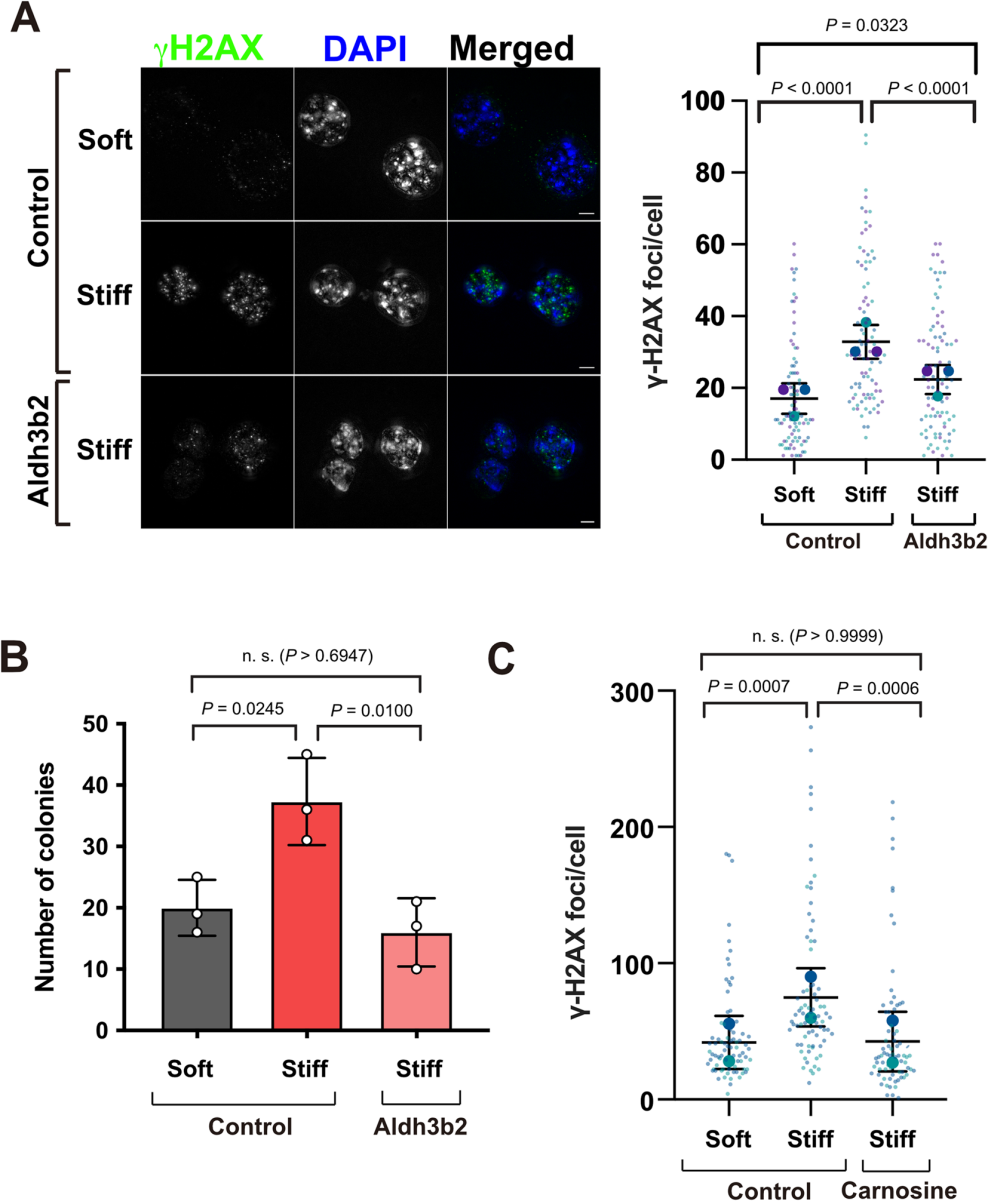
Re-expression of Aldh isozymes abrogates induction of DNA damage and transformation in MECs due to ECM stiffness. **A.** WT EpH4 cells or those stably overexpressing *Aldh3b2* grown in soft or stiff 3D ECM as indicated. Left panel shows representative IF images of EpH4 cells stained for γH2AX (phospho-Ser139), (scale bars = 5 mm). Right panel shows quantification of γH2AX (phospho-Ser139) foci/cell. Mean ± SD, *n* = 3 per condition from independent experiments. Small data points are foci from individual nuclei in each independent experiment, large data points represent mean number of foci for each independent experiment, calculated from 30 cells/condition. Two-way ANOVA with Tukey’s post-hoc test. **B. **Number of colonies formed in soft agar following culture in soft or stiff 3D ECM for seven days. Mean ± SD, *n* = 3 per condition from independent experiments, each performed in triplicate. Data points represent mean number of colonies from triplicates for each independent experiment. One-way ANOVA with Tukey’s post-hoc test. **C.** Quantification of γH2AX foci in EpH4 cells following culture in soft or stiff 3D ECM with or without carnosine. Mean ± SD, *n* = 2 per condition from independent experiments. Small data points are foci from individual nuclei in each independent experiment, large data points represent mean number of foci for each independent experiment, calculated from 20-55 cells/condition. Two-way ANOVA with Tukey’s post-hoc test.

To determine whether downregulation of Aldh activity was sufficient to induce DNA damage, we generated an EpH4 cell line stably expressing Aldh3b2 under the constitutive EF1α promoter. We chose Aldh3b2 as this was the most significantly downregulated Aldh isoform identified in the RNAseq data, and is similar in function to Aldh3b1, the most downregulated Aldh isoform observed in the proteomic dataset. We seeded these cells into stiff MAG hydrogels and quantified γH2AX foci compared to wildtype (WT) cells. When cultured in stiff MAG, Aldh3b2-expressing EpH4 cells had significantly fewer γH2AX foci than WT cells (Fig. 5A). Furthermore, Aldh3b2 overexpressing EpH4 cells passaged in stiff MAG formed fewer colonies in soft agar (Fig. 5B). Treatment of WT EpH4 cells with carnosine also reduced the number of γH2AX foci in cells grown in stiff MAG to a level similar to that in cells grown in soft MAG (Fig. 5C). Together, these data indicate that culture of MECs within a stiff 3D ECM result in downregulation of *Aldh* genes, and the subsequent accumulation of reactive aldehydes contributes to increased DNA damage and transformation.

### YAP and RhoA activation regulate acini morphology in response to ECM stiffness

**Fig. 6:**
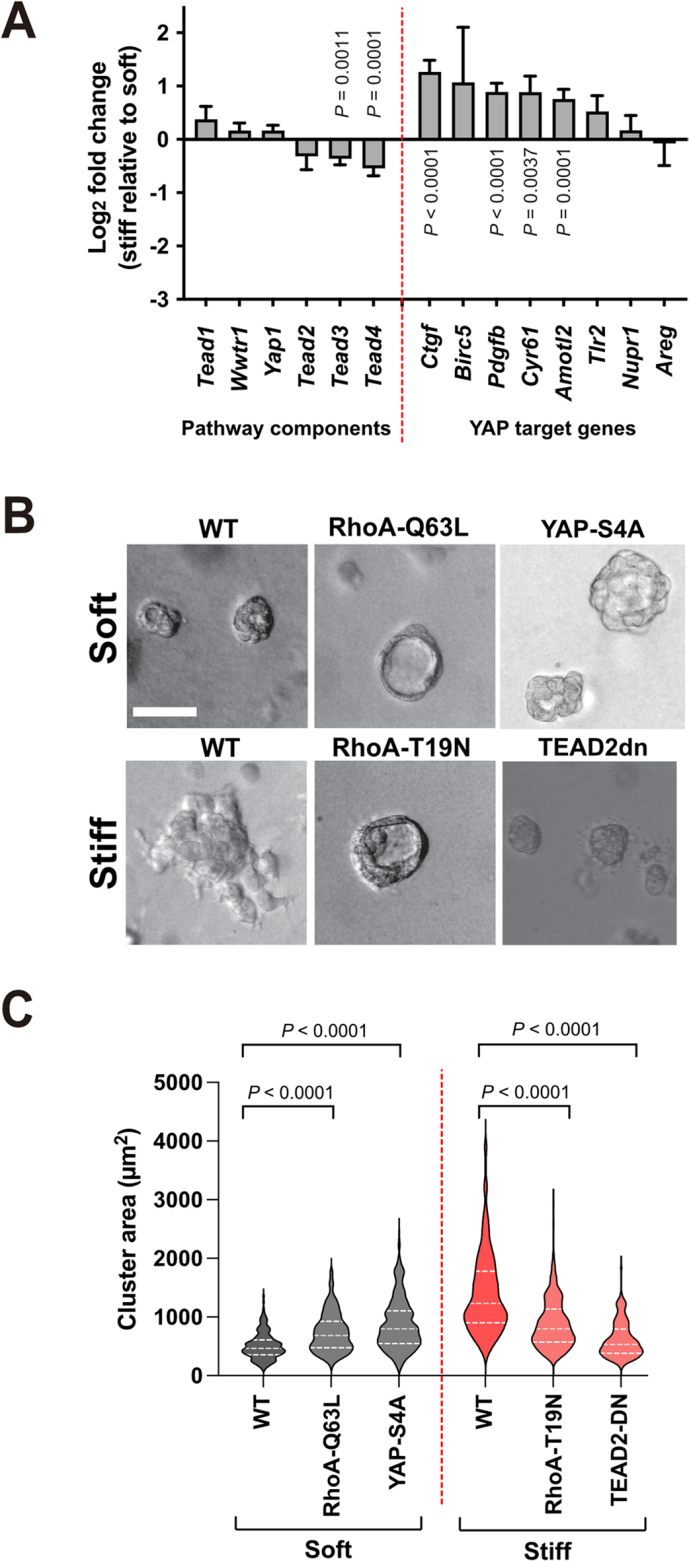
ECM stiffness-induced YAP and RhoA activation regulates acini morphology. **A.** Log_2_ fold-change in expression of genes involved in the YAP/TAZ signalling pathway in EpH4 cells grown in the stiff condition relative to soft, as determined by RNAseq. Error bars represent SEM (*n* = 3). Statistical significance was determined using DESeq2. **B.** Representative brightfield images of EpH4 acini, WT, or those stably expressing YAP-4SA or TEAD2-DN, or expressing either RhoAQ63L or RhoAT19N, were grown in soft or stiff 3D ECM, as indicated for 10 days (scale bar, 50mm). **C.** Quantification of EpH4 acinar area from **B**. Acinar area calculated from three independent experiments, using 40-100 cells/condition. Two-way ANOVA with Tukey’s post-hoc test. Quantification of gH2AX (phospho-Ser139) foci in EpH4 cells, WT or stably expressing YAP-4SA or TEAD2-DN, following 24 hours of 3D culture. Mean ± SD, *n* = 3 per condition from independent experiments. Small data points are foci from individual nuclei in each independent experiment, large data points represent mean number of foci for each independent experiment, calculated from 30 cells/condition. Two-way ANOVA with Tukey’s post-hoc test.

We next sought to determine the signalling mechanism by which mechanical stiffness contributed to increased aldehyde-induced DNA damage. The RNAseq and proteomic data obtained from EpH4 cells grown in soft and stiff 3D MAG indicated changes in mechanosignalling through the small GTPase Rho (Fig. 3C and Fig. 4B). RhoA is a key regulator of mechanical response in cells and previous studies have implicated it in driving MEC transformation^14^. YAP/TAZ (Yes-associated protein/Transcriptional coactivator with PDZ-binding motif) is another established mechanosensitive signalling pathway with known links to proliferation and tumourigenesis^27^. The RNAseq data showed that there was upregulation of several known YAP/TAZ target genes in cells cultured in stiff MAG, including connective tissue growth factor (*Ctgf*) (Fig. 6A). We therefore asked if manipulating RhoA or YAP altered the response of EpH4 cells to 3D MAG stiffness.

To investigate the role of RhoA signalling we generated EpH4 cell lines stably expressing two variants: RhoA-Q63L, which exhibits impaired GTP hydrolysis, resulting in sustained RhoA activity in response to a stimulus; and RhoA-T19N, which attenuates RhoA activity by acting as a dominant negative. To activate YAP we generated an EpH4 line expressing YAP-4SA. This has four inhibitory serine phosphorylation sites substituted to alanine, resulting in constitutive YAP activation^28^. To inhibit YAP signalling we expressed a dominant negative TEAD2 (TEAD2-DN) lacking the DNA binding domain, which blocks YAP-dependent transactivation^29^. We compared the effect of activating RhoA or YAP signalling in a soft 3D MAG, and inhibiting them in a stiff hydrogel.

WT EpH4 cells and those expressing either RhoA-Q63L or YAP-S4A were seeded into soft MAG hydrogels. Acini were grown for 10 days and then their size quantified (Fig. 6B, C). In soft MAG, expression of either RhoA-Q63L or YAP-4SA significantly increased EpH4 acinar size compared to WT cells, although not to the extent seen with acini in a stiff 3D matrigel alginate. In contrast, inhibiting YAP signalling by expressing TEAD2-DN inhibited the increased acinar size in cells grown in stiff MAG (Fig. 6B, C). Similarly, the dominant negative RhoA-T19N also significantly reduced the effect of a stiff MAG hydrogel on acinar size.

These data suggest that both YAP and RhoA may have a role in some of the morphological changes associated with the mechanosignalling response of MECs in MAG.

### RhoA drives reactive aldehyde accumulation and DNA damage in a stiff 3D ECM

**Fig. 7:**
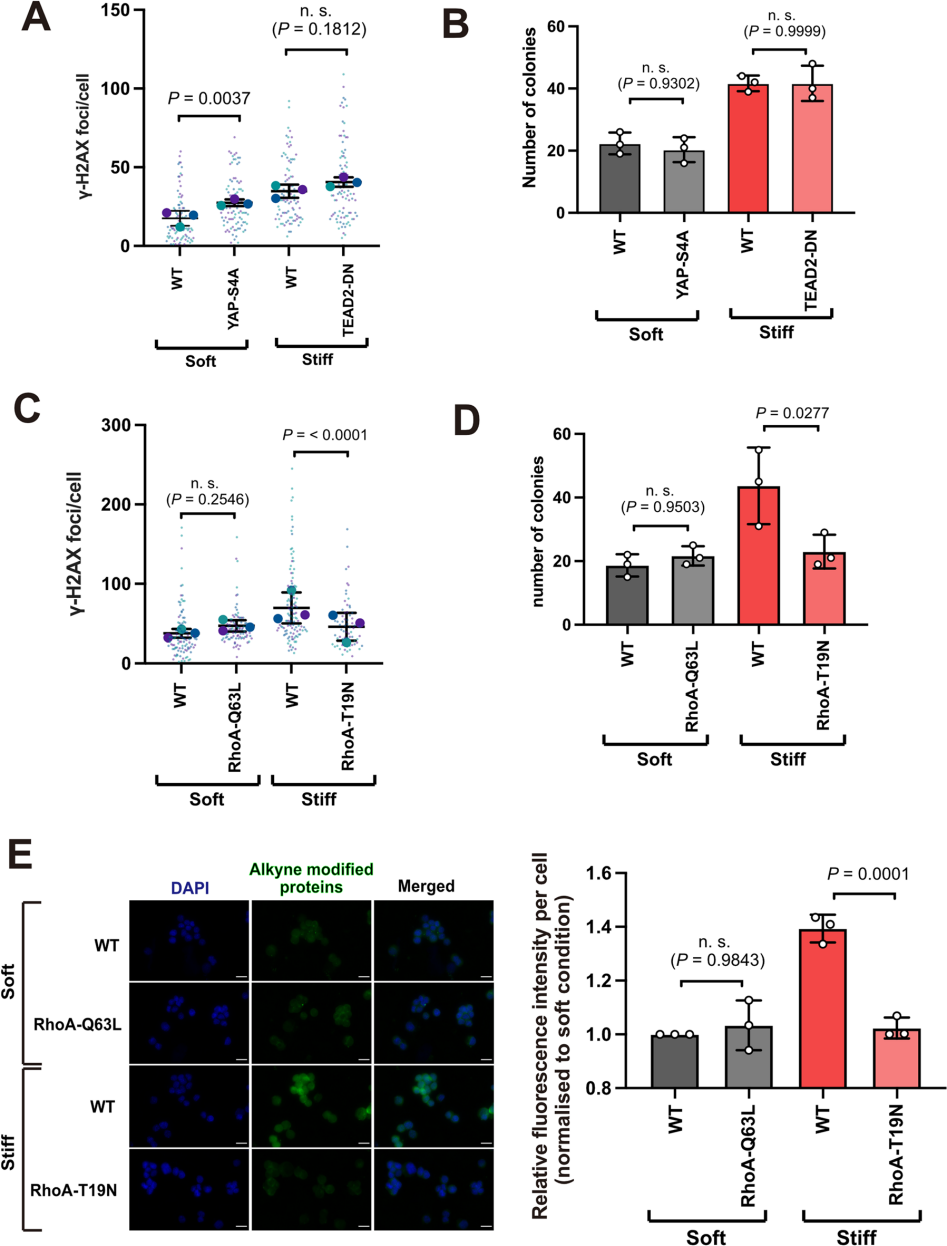
Activation of RhoA signalling drives reactive aldehyde accumulation and DNA damage in cells cultured in stiff ECM. **A.** Quantification of γH2AX (phospho-Ser139) foci in EpH4 cells, WT or stably expressing YAP-4SA or TEAD2-DN, following 24 hours of 3D culture. Mean ± SD, *n* = 3 per condition from independent experiments. Small data points are foci from individual nuclei in each independent experiment, large data points represent mean number of foci for each independent experiment, calculated from 30 cells/condition. Two-way ANOVA with Tukey’s post-hoc test. **B.** Number of colonies formed in soft agar following culture of EpH4 cells, WT or stably expressing YAP-4SA or TEAD2-DN in soft or stiff 3D ECM for seven days. Mean ± SD, *n* = 3 per condition from independent experiments, each performed in triplicate. One-way ANOVA with Tukey’s post-hoc test. **C.** Quantification of γH2AX foci (phospho-Ser139) in EpH4 cells, WT or expressing either RhoAQ63L or RhoA-T19N, following 24 hours of 3D culture. Mean ± SD, *n* = 3 per condition from independent experiments. Small data points are foci from individual nuclei in each independent experiment, large data points represent mean number of foci for each independent experiment, calculated from 15-50 cells/condition. Two-way ANOVA with Tukey’s post-hoc test. **C. **Number of colonies formed in soft agar by EpH4 cells, WT or expressing either RhoAQ63L or RhoA-T19N, following 7 days culture in either soft or stiff 3D ECM as indicated. Mean ± SD, *n* = 3 per condition from independent experiments, each performed in triplicate. Data points represent mean number of colonies from triplicates for each independent experiment. One-way ANOVA with Tukey’s post-hoc test. **E. **Representative images of EpH4 cells, WT or expressing either RhoAQ63L or RhoA-T19N, treated and stained with Click-iT™ Lipid Peroxidation Imaging Kit after culture in gels of different stiffnesses. Left panel shows representative immunofluorescence images of cells following treatment and staining as indicated (scale bar, 20mm). Right panels shows immunofluorescence quantification. (scale bars, 20mm). Mean ± SD, *n* = 3 per condition from independent experiments. Data points represent the median fluorescence intensity for each independent experiment normalised to the median value for the soft, untreated condition (>500 cells/condition). One-way ANOVA with Tukey’s post-hoc test.

As both YAP and RhoA altered stiffness induced acinar size, we next asked whether they also influenced DNA damage. The stable EpH4 lines were seeded into soft or stiff MAG hydrogels, and quantification of γH2AX foci carried out as in Fig. 2. Expression of the active YAP-4SA was sufficient to increase the number of γH2AX foci in cells cultured within soft MAG, suggesting the activation of YAP signalling could promote genomic damage (Fig. 7A). However, inhibiting YAP in the TEAD2-DN expressing cells did not reduce the elevated level of DNA damage seen in stiff MAG hydrogels (Fig. 7A). Furthermore, neither activation nor inhibition of YAP signalling altered the number of EpH4 colonies formed in soft agar following culture in 3D MAG hydrogels (Fig. 7B). Thus, the increased DNA damage observed in cells cultured in stiff hydrogels and their subsequent transformation was not dependent upon YAP signalling.

We then looked at EpH4 cells expressing either RhoA-Q63L, which showed a small but significant increase in acinar size compared to WT cells, or RhoA-T19N which formed smaller acini in stiff 3D-cultures compared to WT cells (Fig. 6C). Stable lines expressing each variant were quantified for γH2AX foci number and colony formation in soft agar. RhoA-Q63L expression in soft MAG was not sufficient to drive a significant increase in DNA damage or soft agar colony formation (Fig. 7C, D). However, expression of RhoA-T19N attenuated both the accumulation of DNA damage, seen *via* reduced number of γH2AX foci, and decreased colony formation in soft agar (Fig. 7C, D). Indeed, DNA damage and colony formation in MEC within a stiff MAG were reduced by RhoA-T19N to the levels observed in WT cells grown in soft hydrogels.

We next asked whether RhoA-dependent DNA damage was linked to reduced Aldh activity. As several *Aldh* genes were potentially downregulated, we assessed the levels of alkyne-modified protein in LAA-treated cells, which is indicative of reactive aldehyde accumulation and therefore Aldh isoform independent. WT, RhoA-Q63L and RhoA-T19N expressing EpH4 cells were cultured in soft and stiff MAG as above. We compared single cell levels of alkyne-modified protein in WT cells and those expressing RhoA-Q63L in soft, and RhoA-T19N in stiff (Fig. 7E). As with DNA damage and colony formation, cells expressing RhoA-Q63L did not exhibit an increase in alkyne-modified protein compared to WT. However, inhibition of RhoA activity through expression of RhoA-T19N reduced alkyne-modified protein levels to those observed in WT cells in soft ECM.

These results demonstrate a RhoA-dependent mechanism of DNA damage accumulation via downregulation of Aldh, and decreased clearance of reactive aldehyde species, when EpH4 cells are cultured within a stiff 3D hydrogel. Taken together, these findings propose a mechanism by which increased ECM may lead to genomic damage required cancer initiation.

## Discussion

Understanding the mechanistic basis of how defined risk factors promote carcinogenesis is important for mitigating their effects. High MD is a significant risk factor for developing breast cancer. Indeed, some estimates are that high MD may account for up to one third of breast cancers^11^. High MD is associated with increased peri-ductal stromal stiffness and thus altered mechanotransduction^10,12^. Given there is now a general understanding that most aspects of cell behaviour are governed by mechanotransduction^30^, we here examined the effects of increased ECM stiffness on MECs using a mechanically-tuneable 3D hydrogel system^13^. Analysis of global gene expression induced by increased ECM stiffness identified significant changes within multiple metabolic pathways. We found that decreased cellular Aldh isozyme expression contributed to the accumulation of reactive aldehydes, leading to increased DNA damage and acquisition of anchorage-independent growth. Our results suggest that mechanotransduction-induced changes in aldehyde metabolism leading to DNA damage may be a contributing factor for breast cancer risk associated with high MD.

Mechanical cues from the ECM have been linked to cellular metabolism^22,23^. Mechanosignalling changes in metabolic processes occur through both transcriptional and post-translational regulation. Here we found global changes in metabolic pathways at the transcriptional level coordinated through differences in ECM stiffness in 3D. Some of the most significant changes were in oxidoreductase pathways, with members of the Aldh isozyme family being highly downregulated at both the transcript and protein level. The Aldh family are essential in the detoxification of endogenous reactive aldehydes by catalysing their oxidation to carboxylic acids. Failure to remove aldehydes leaves them free to react with proteins and DNA. The reaction of these aldehydes with DNA results in the formation of base adducts which ultimately lead to DNA strand breaks during DNA replication, a major source of mutations^24^. Most of the work relating to Aldh expression and breast cancer centres on *Aldh1A1* as a stem cell marker^25,31^. In breast cancer, ALDH1A1 has been implicated in the acquisition of drug resistance, associated with poor prognosis^25,32^. We did not see changes in *Aldh1A1* expression. Instead, we suggest a novel, mechanosensitive mechanism by which lowered Aldh activity results in DNA damage through increased endogenous aldehydes. Clinical studies have shown increased levels of urinary malodialdehyde in women with high MD^33,34^. Furthermore, increased DNA damage is also seen in primary tissue samples taken from women with high MD^35^.

Many signalling pathways have been described to regulate mechanosensitive cell behaviour. We identified both Rho and YAP signalling being regulated in MEC acini within a stiffer 3D MAG hydrogel. Although YAP activity appears to influence MEC acinar morphology and size, inhibiting its signalling did not alleviate DNA damage in stiff 3D ECM. Studies in 2D have identified that YAP nuclear translocation is dependent on perinuclear stress fibre formation resulting in the widening of nuclear pores, and disruption of this process leads to reduced YAP nuclear localisation^36–38^. A study assessing YAP dynamics in MECs in 3D found that stress fibres failed to form in mammary acini in both soft and stiff 3D hydrogels, resulting in cytosolic localisation of YAP regardless of stiffness^39^. Furthermore, both primary and immortalised breast cancer cells in 3D show a lack of YAP nuclear localisation^40^. However, YAP-mediated mechanotransduction in 3D can be enhanced by oncogenic KRAS and HER2, increasing the sensitivity of the cells to changes in their microenvironment^41^. This may suggest that YAP might have more impact in cooperation with specific oncogenic mutations.

The role of Rho in mechanosignalling is well documented^42^. In the context of breast cancer, disruption of Rho signalling can reverse the transformed phenotype observed in MECs in 3D collagen hydrogels upon ECM stiffening^14^. Here we suggest a role for Rho-mediated mechanosignalling in driving the accumulation of reactive aldehyde species, resulting in DNA damage. The mechanisms by which RhoA influences aldehyde metabolism remain unknown. However, it is possible that they may occur through the influence of RhoA on transcriptional regulators. RhoA-dependent SRF transcriptional activity has recently been shown to facilitate the upregulation of glutamine metabolism in MECs expressing oncogenic Myc, suggesting an important role for RhoA in cancer cell metabolism^43^. Furthermore, studies have established the negative regulation of SREBP1 by Rho-stimulated actomyosin contractility and regulation of lipid synthesis pathways^23,44^. In agreement, we found that genes involved in fatty acid metabolism exhibit some of the largest fold changes in stiff compared to soft 3D MAG, providing a potential candidate for how RhoA may influence metabolism in this system. Dissecting downstream RhoA interacting partners in different 3D-hydrogel conditions may establish which signalling pathways interact differently between soft and stiff conditions^45^.

In conclusion, we demonstrate a novel, RhoA-mediated regulation of oxidoreductase pathways leading to the accumulation of reactive aldehydes in stiff 3D matrices. These results provide a potential mechanism by which the increased ECM stiffness of the periductal stroma in women with high MD drives the genetic changes required for breast cancer initiation. Therefore, these data provide a possible explanation as to how high MD confers an increase in breast cancer risk.

## Methods

### Cell culture

EpH4 cells were derived from spontaneously immortalised mouse MECs^17^. They were originally purchased from ATCC and cultured as we have previously described^18^ in DMEM-F12 with 10% FBS (v/v), supplemented with 5 µg/mL insulin and 1% penicillin/streptomycin (v/v). For differentiation assays, growth media was removed, and cells were washed briefly in PBS. Cells were then cultured in differentiation medium (DMEM-F12, 10% FBS (v/v), 5 µg/mL insulin, 0.5 µg/mL hydrocortisone, 1% penicillin/streptomycin (v/v) and 3 µg/mL ovine prolactin). HEK-293T cells for lentiviral generation were cultured in DMEM supplemented with 10% FBS (v/v).

### Generation of Matrigel/Alginate (MAG) hydrogels

MAG gels were generated as described previously^13^. Briefly, 9.8 mg/mL Matrigel (Corning − 354234) was combined with 25 mg/mL Pronova SLG 100 sodium alginate (Novamatrix − 4202101) in a 2:1 ratio on ice. 0.5–1 × 10^5^ cells were then mixed in with each gel mixture. 50 µL 0 mM (blank DMEM-F12), 2.4 mM or 24 mM CaSO_4_ slurry was added to the gels to generate soft, stiff, and very stiff gels respectively. This was done by placing the CaSO_4_ slurry and 200 µL Matrigel/Alginate gel into separate 1 mL syringes, connected via a female-female Luer Lock coupler (Sigma − 21015), and mixing through 4 syringe pumps, before ejecting the final gel into a 24 well plate, pre-coated with 40–50 µL of 9.8 mg/mL Matrigel. Gels were set for 30 min at 37 °C in a humidified incubator, prior to the addition of EpH4 complete growth media. Unstained cells and acini were imaged using a Zeiss Axiovert 40 CFL brightfield microscope with either a LD A-plan 20x, 10x or 5x objective lens. Images were captured via Micro-Manager v1.4 software.

To extract acini from hydrogels, assay medium was removed, wells rinsed with sterile, 1X PBS, and 1X Trypsin-EDTA added and pipetted to manually break the gels. Trypsin-cell mixtures were incubated for 5 min at 37 °C. Digested mixes from replicate wells were pooled and spun at 1000 x g for 5 min at 4 °C. Cell pellets could be resuspended for genomic DNA extractions, RNA extractions, soft agar colony formation assays, or for cytospinning.

### Nanomechanical testing of Matrigel/Alginate (MAG) hydrogels

Nanomechanical testing was done using a Hysitron Biosoft nanoindenter (Bruker-Hysitron, Minneapolis MN, USA) fitted with 800 μm diameter spherical sapphire probe. Cell-free MAG gels were prepared as described above, but with 500 µL volumes in 24-well plate, coated with a 50 µL Matrigel base to ensure adhesion to the plate. Gels were set for 30 min in an incubator at 37^o^C prior to the addition of 1.5 mL of media and further incubated for 24 hrs to ensure hydration and equilibrium swelling. Gels were measured in hydrated state, with a minimal volume of medium (0.5 mL). The nanoindenter was used to obtain elastic modulus values using force relaxation, where the probe was set into the gel and left for 30 min to allow the gel to relax before each test. The load profile used consisted of a 25 μm delta displacement with a 200 s hold time. Each gel was tested in triplicate and the result averaged. Load vs. Time curves were analysed using TriboIQ analysis software (Bruker-Hysitron Minneapolis, MN, USA) using force-relaxation analysis (Eq. 1) and elastic modulus calculated using Hertzian contact equation for spherical contact (Eq. 2).

1 $$\:P=\:{{\upgamma\:}}_{\text{o}}+\:{A}_{1}{e}^{-\frac{t}{T1}}+\:{A}_{2}{e}^{-\frac{t}{T2}}\text{}\text{}\text{}\text{}$$

where$\:\:{{\upgamma\:}}_{\text{o}}$ represents the load at equilibrium, $\:A$ is the amplitude, $\:t$ is time variable and $\:T$ is time constant.

2 $$\:{E}_{\infty\:}=\frac{3}{4\sqrt{R}}\:\frac{1-{v}^{2}}{{h}^{\frac{3}{2}}}\:{{\upgamma\:}}_{\text{o}}$$

where elastic moduli ($\:{E}_{\infty\:\:}$) is calculated using an assumed Poisson ratio ($\:v$) of 0.4. $\:R$ is radius and $\:h$ indicates contact deformation.

### Plasmids

A pEGFP-YAP-4SA was then generated by introducing S61A, S109A, S164A and S381A mutations into pEGFP-YAP by site-directed mutagenesis. pCDH-TEAD2-DN-GFP-H2B-RFP was generated by PCR amplification of the C-terminal YAP-binding domain of TEAD2 from the pCMX-Gal4-TEAD2 construct (a gift from Kunliang Guan, Addgene: 33107)^46^. This amplified TEAD fragment was then subsequently cloned into pCDH-GFP-H2B-RFP. RhoA-T19N was a gift from Patrick Caswell. A pCDH-BFP-RhoA-T19N construct was generated from this by PCR amplification and subsequent insertion of the RhoA-T19N into pCDH-EF1α-tagBFP. The pCDH-BFP-RhoA-Q63L construct was generated from the pCDH-BFP-RhoA-T19N construct through N19T and Q63L mutation of the RhoA by site-directed mutagenesis. All constructs were verified by Sanger sequencing at GATC (Eurofins).

### Lentivirus generation and transduction of EpH4 cells

HEK-293T cells were transfected overnight with 3 µg packaging vector pMD2.G, 4.5 µg packaging vector psPax2, and 6 µg of the plasmid containing the gene of interest using 1X PEI reagent. Transfection media was then discarded and replaced with media containing 1% (v/v) sodium butyrate (Merck-Millipore – 19–137) for 8 hrs. Cells were then returned to standard growth media for 36 hrs. Following this, media containing lentivirus was harvested, and filtered through a 0.45 µm filter. Lentivirus was then precipitated through addition of 4X PEG lentivirus precipitation solution (0.05 M PEG-800, 1.2 M NaCl in 1X PBS, pH 7.4) and incubation for 12 to 72 hrs at 4 °C. Lentivirus was then pelleted by centrifugation at 1500 x g for 30 min. The supernatant was then removed and centrifuged for a further 5 min. The two pellets were then combined and resuspended in a small amount of growth media and stored at −80 °C prior to transduction.

For transduction, 1 × 10^4^ EpH4 cells were cultured for 24 hrs in EpH4 growth media. The next day, media was changed to EpH4 growth media containing 0.1% (v/v) polybrene infection/transfection reagent (Merck-Millipore – TR-1003-G), and lentivirus was added to the cells for 24 hrs. Cells were then washed three times in complete growth media and once in PBS and cultured for 2 weeks in growth media prior to sorting of transduced cells via FACS.

### Quantitative reverse transcription PCR (RT-qPCR)

RT-qPCR gene expression analysis was performed using either TaqMan Fast Advanced Master Mix or Fast SYBR Green Master Mix according to the manufacturer’s protocols on a StepOnePlus qPCR machine (Applied Biosystems). Fluorescence was used to calculate 2-(ΔΔCT) for statistical analysis using the ΔΔCT method. Values obtained for GAPDH were used to normalise values for the genes of interest. Primers used for the SYBR Green method are as follows: GAPDH: forward – 5’-GGTGAAGGTCGGAGTCAACGG-3’, reverse 5’-GAGGTCAATGAAGGGTCATTG-3’; Sox9 : forward – 5’-GAGCCGGATCTGAAGAGGGA-3’, reverse – 5’-GCTTGACGTGTGGCTTGTTC-3’; Egr2: forward – 5’-GCCAAGGCCGTAGACAAAATC-3’, reverse – 5’- CCACTCCGTTCA TCTGGTCA-3’; Snail2: forward 5’- AAGATGCACA TCCGAAGCCA-3’, reverse 5’- CTCTTGGTGCTTGTGGAGCA-3’. Probes used for the TaqMan method are as follows: GAPDH Mm99999915_g1; Csn Mm04207885_m1; PrlR Mm00599957_m1; Krt5 Mm01305291_g1; Krt14 Mm00516876_m1; Vim Mm01333430_m1; Elf5 Mm00468732_m1; Krt18 Mm01601704_g1; Cdh1 Mm01247357_m1. Primers for Sox9, EGR2 and Snail2 were from^47^.

### RNA sequencing

Polyadenylated mRNA was purified from 1 µg total RNA using poly-T oligo magnetic beads. Quality and integrity of total RNA were confirmed using a 2200 TapeStation (Agilent Technologies). mRNA libraries were then generated using TruSeq Stranded mRNA Assay (Illumina inc.) according to the manufacturer’s protocol. Multiplex libraries were then generated using adaptor indices and pooled prior to cluster generation using a cBot instrument. The loaded flow-cell was then paired-end sequenced (76 + 76 cycles, plus indices) on an Illumina HiSeq4000 instrument. Unmapped paired-end sequences from an Illumina HiSeq2500 sequencer were tested by FastQC, (http://www.bioinformatics.babraham.ac.uk/projects/fastqc/). Sequence adapters were removed, and reads were quality trimmed using Trimmomatic^48^. The reads were mapped against the reference mouse genome (mm10/GRCm38) and counts per gene were calculated using annotation from GENCODE M2 (http://www.gencodegenes.org/) Tophat2^49^ and HTSeq^50^. Normalisation, Principal Componenents Analysis, and differential expression were calculated with DESeq2^51^.

### Gene ontology (GO) analysis for RNAseq

Over-represented GO terms of significantly changing genes (adjusted *p* < 0.05 and fold change ± 2) were calculated by the R package TopGO^52^. GO terms with adjusted p-values < 0.05 were considered significant^53^. For the GO-gene bipartite graph, the significantly changing genes were submitted for to GO over-representation analysis using the R package ClusterProfiler^54^. ClusterProfiler results were simplified using semantic similarity^55^ to cluster terms with similarity score > 0.7, with the representative term taken from each cluster with the lowest adjusted p-value. GO terms with adjusted p-values < 0.05 were considered significant.

### Mass spectrometry

1.5 × 10^6^ EpH4 cells per condition were cultured in soft and stiff MAG gels for 10 days. Gels were depolymerised by incubation with Corning Cell Recovery Solution for 1–2 hrs, allowing extraction of cells by centrifugation at 500 RCF for 5 min. Pellets were washed once via resuspension in alginate wash buffer (150 mM NaCl, 55 mM sodium citrate, 30 mM EDTA in ddH_2_O; pH 6.8), prior to centrifugation at 500 x g for 5 min. Cells were then washed 3 times in 10 mL 1X PBS, prior to centrifugation at 500 RCF for 5 min. Cells were then lysed directly in 1X S-Trap™ lysis buffer (5% SDS, 50 mM TEAB, pH 7.6). Samples were sonicated to shear genomic DNA and centrifuged at 20,000 x g to clarify the lysates. Protein concentration was determined by BCA assay (Pierce) according to the manufacturer’s protocol. Protein lysates were prepared for mass spectrometry using S-Trap^™^ methodologies according to the manufacturer’s protocol. Briefly, proteins were reduced using 500 mM DTT, cooled to room temperature, and then alkylated using 500 mM iodoacetamide. H_3_PO_4_ was then added to acidify the samples, prior to washing and the addition of 6 volumes of S-Trap™ binding buffer (90% MeOH, 100 mM final TEAB, pH 7.1). Samples were then added to the S-Trap™ spin column. Samples were bound to the column through centrifugation at 4000 x g for 1 min. Samples were then washed 3 times in S-Trap™ binding buffer. Proteins were then digested in 2 µg trypsin diluted in 75 µL S-Trap lysis buffer at 47 °C for 1 h. Peptides were then eluted in digestion buffer, and further eluted using 0.1% formic acid. Peptides were then desalted using POROS R3 beads and eluted as previously published^56^, and lyophilised via vacuum centrifugation prior to resuspension in injection buffer (5% ACN, 0.1% FA in water). Samples were analysed using the QE-HF mass spectrometer by the BioMS core facility at the University of Manchester.

### Mass spectrometry analysis

Raw data were processed using MaxQuant (v1.6.14.0, available from Max Planck Institute of Biochemistry)^57^. Features were identified using default parameters in MaxQuant, then searched against the murine proteome (obtained from Uniprot, June 2021). Oxidation of methionine (M) & proline (P), phosphorylation of serine (S), tyrosine (Y) & threonine (T) and acetylation of protein N-terminus were set as variable modification, whereas carbamidomethyl (C) was set as a fixed modification. Peptide quantitation was performed using label-free quantification (LFQ), using only unmodified, unique peptides and with ‘match between runs’ enabled. Statistical analysis was performed using MSqRob^58,59^. LFQ data was normalised by means of the median of peptide intensities. Condition (stiff or soft) was treated as a fixed effect. Peptide sequence and technical replicate were treated as random effects. Peptides belonging to contaminant protein lists (annotated by MaxQuant) or proteins with fewer than 2 peptides were excluded from analysis. For annotation of individual peptides ECM/non-ECM status, the peptide table generated by MaxQuant was screened against the MatrisomeDB, a curated database of ECM proteins^60,61^. ECM and non-ECM peptides were then processed separately through MSqRob. Sequence alignment of Aldh3b1 and Aldh3b2 was performed using the Clustal Omega server^62^. GO analysis was performed using PANTHER GO (Version 16.0) Enrichment Test (Release 2021-02-24) against GO database DOI: 10.5281/zenodo.5228828 (Released 2021-08-18)^63^. GO terms were considered significant with a False Discovery Score of < 0.05.

### ClueGO enrichment analysis of RNA-seq and proteomic datasets

GO enrichment analysis of RNA-seq and proteomic datasets was performed using ClueGO (version 2.5.9), a Cytoscape plug-in which generates functionally grouped GO networks^64,65^. GO analysis was restricted to differentially expressed genes and proteins with an adjusted p-value of < 0.05 and genes with an absolute fold change of ± 1.5. GO terms with an adjusted p-value of < 0.05 were considered significant. GO terms were clustered into functional groups based on term-term association using kappa statistics (kappa score ≥ 0.4). The fusion functionality within ClueGO was used to reduce redundancy between similar GO terms. ClueGO accessed February 2023.

### Immunofluorescence microscopy

Single cells isolated from gels were resuspended in 1X PBS, 50 µL cytospun at 400 RPM for 5 min onto polysine slides using a Shandon Cytospin 2 (Thermo Scientific) and fixed onto the slides with 4% PFA. Fixed cells were permeabilized using 0.5% Triton-X, then washed in 1X TBS. The cells were then blocked using 3% BSA in TBS for at least 1 h before addition of the primary antibody in antibody dilution buffer (1% horse serum (v/v), 0.05% Tween 20 (v/v), 0.2% Triton-X-100 (v/v), and 0.05% sodium azide (v/v) in 1X PBS. Cells were incubated with primary antibody for at least 1 hr at room temperature, or overnight at 4 °C. The primary antibody solution was then removed and cells washed several times with 1X TBS before addition of the secondary antibody in antibody dilution buffer. Cells were incubated with secondary antibody solution for 1 h at room temperature, then washed several times with 1X TBS. Cells were then incubated with DAPI (0.1 µg/ml) for 10 min, then washed several times in 1X TBS and once in ddH_2_0 and mounted in DAKO fluorescent mounting medium (Agilent Technologies). Images were taken using a Zeiss Imager M2 fluorescence microscope with an 63x (NA 1.4) Plan Apochromat objective and a Hamamatsu ORCA-ER Digital Camera using ImageJ. To quantify γH2AX foci, z-stack images were taken on an Olympus Ix51 microscope with a 100x PlanAPO NA 1.4 objective, deconvolved and converted into a maximum projection. Images were analysed using cell profiler, using DAPI to define individual nuclei, and the number of foci in each quantified.

For confocal imaging of intact acini, following their isolation as described above, acini were pelleted at 70 x g for 3 min at 4^o^C and fixed for 45 min in 4% (wt/vol) PFA. Acini were washed in PBS at 4^o^C before blocking in acini wash buffer (AWB – PBS, pH7.4, 0.1% Triton X100, 0.2% bovine serum albumin) and transferral to low-adherence 24 well plates. Acini were incubated with primary antibody diluted in AWB overnight on a low-speed orbital shaker (< 100 rpm) at 4^o^C. Acini were washed 3 times in AWB at 4^o^C for 2 h before adding the appropriate 2x secondary antibodies in AWB overnight at 4^o^C. Acini were washed once for 10 min in AWB, then incubated with 2x DAPI in AWB for a further 10 min. Acini were then washed a further 3 times in AWB at 4^o^C for 2 h. After the final wash, organoids were diluted in PBS and transferred to the wells of a 6 well plate for imaging. Images were taken using a Leica TCS SP8 AOBS upright confocal microscope using a 60x/0.9 HCX Apo U-V dipping lens. The confocal settings were: pinhole 1 airy unit, scan speed 600 Hz bidriectional, formation 1024 × 1024. Images were collected using hybrid and Photon Multiplying Tube Detectors with the following detection mirror settings: DAPI 410–475 nm; Alexa-488 505–580 nm; Alexa-594 605–750 nm, using the white light laser with 488 nm (20%), 594 nm (10%) and 633 nm (10%) laser lines respectively. Images were captured through sequential scanning. Acquired images were processed using ImageJ.

### Alkyne-modified linoleic acid (LAA) assay

Cells were grown in soft and stiff MAG hydrogels for 24 h. Five hrs prior to extraction from the gels, 50 µM LAA was added to the media. Cells were then extracted, fixed in 4% PFA, washed once in PBS, and cytospun onto polysine slides. Staining for lipid peroxidation was then carried out using a Click-iT Lipid Peroxidation Imaging Kit – Alexa Fluor 488 (Invitrogen) according to the manufacturer’s protocol and mounted with DAKO fluorescent mounting medium (Agilent Technologies). Images were acquired on a 3D-Histech Pannoramic-250 microscope slide-scanner using a 40x/0.95 Plan Apochromat objective (Zeiss) and the DAPI and FITC filter sets. Quantification of fluorescence intensity was completed using QuPath (0.2.0-m8)^66^ and images were exported and processed in Fiji^67^.

### EdU Click-iT assay

Click-iT EdU labelling, Alexa Fluor 488 dye (Thermo Fisher Scientific), was performed as per the manufacturer’s protocols. Cells were labelled with the EdU solution for 5 hrs and then extracted from hydrogels, cytopsun and fixed as described a above. Cells were incubated with 30 µL Click-iT Edu Alexa Fluor 488 reaction cocktail (made as per the manufactures guidelines) for 30 min, washed and incubated with 0.1 µg/mL DAPI for 10 min. Cells were mounted and imaged as above.

### Colony-formation assay

1 mL of molten 0.8% low gelling agarose solution was added to the bottom of each well of a 6 well plate and allowed to set for 20 min at room temperature. Following this, EpH4 cells grown for 7 days in rBM/Alginate gels were extracted and 1 × 10^4^ seeded inside 0.4% low-gelling agarose solution and cast on top of the base layer. Plates were then placed on ice to cool until set. 1 mL of growth media was then added to each well and cells were incubated for 21 days at 37 °C and 5% CO_2_ under humidified conditions, with media replaced every 4 days. Following incubation, cells were fixed in 0.005% crystal violet solution in 4% PFA for 1 h. The number of colonies were then counted manually using a Leica DMIL LED Inverted Brightfield microscope.

## Statistical analysis

All statistical analyses were carried out using PRISM 8 for MacOS (version 8.4.3 (471)). Where appropriate, statistical significance was determined by Two-tailed student’s *t*-test, one- or 2-way ANOVA with Tukey’s post-hoc test, or Kruskal Wallis test with Dunn’s multiple comparisons post-hoc test. The test used for each experiment is defined in the figure legends.

## Electronic supplementary material

Below is the link to the electronic supplementary material.

Supplementary Material 1

## Data Availability

The RNAseq datasets generated and/or analysed during the current study are available in the ArrayExpress repository, E-MTAB-15122. The proteomic data sets are available in PRIDE, accession PXD063452. For reviewer access log in to the PRIDE website using the following details: Project accession: PXD063452, Token: OKFt0N4FB7Iv.

## References

[1] Wirtz, H. R. & Dobbs, L. G. The effects of mechanical forces on lung functions. *Respir Physiol.* **119**, 1–17 (2000). 10701703 10.1016/s0034-5687(99)00092-4

[2] Yamamoto, K. et al. Regulation of cardiomyocyte mechanotransduction by the cardiac cycle. *Circulation* **103**, 1459–1464 (2001). 11245653 10.1161/01.cir.103.10.1459

[3] Wang, N., Butler, J. P. & Ingber, D. E. Mechanotransduction across the cell surface and through the cytoskeleton. *Science* **260**, 1124–1127 (1993). 7684161 10.1126/science.7684161

[4] Muhamed, I. et al. E-cadherin-mediated force transduction signals regulate global cell mechanics. *J. Cell. Sci.* **129**, 1843–1854 (2016). 26966187 10.1242/jcs.185447 PMC4893802

[5] Engler, A. J., Sen, S., Sweeney, H. L. & Discher, D. E. Matrix elasticity directs stem cell lineage specification. *Cell* **126**, 677–689 (2006). 16923388 10.1016/j.cell.2006.06.044

[6] Klein, E. A. et al. Cell-cycle control by physiological matrix elasticity and in vivo tissue stiffening. *Curr. Biol.* **19**, 1511–1518 (2009). 19765988 10.1016/j.cub.2009.07.069 PMC2755619

[7] Wang, H. B., Dembo, M. & Wang, Y. L. Substrate flexibility regulates growth and apoptosis of normal but not transformed cells. *Am. J. Physiology-Cell Physiol.* **279**, C1345–C1350 (2000). 10.1152/ajpcell.2000.279.5.C1345 11029281

[8] Acerbi, I. et al. Human breast cancer invasion and aggression correlates with ECM stiffening and immune cell infiltration. *Integr. Biol. (Camb)*. **7**, 1120–1134 (2015). 25959051 10.1039/c5ib00040h PMC4593730

[9] Levental, K. R. et al. Matrix crosslinking forces tumor progression by enhancing integrin signaling. *Cell* **139**, 891–906 (2009). 19931152 10.1016/j.cell.2009.10.027 PMC2788004

[10] McConnell, J. C. et al. Increased peri-ductal collagen micro-organization May contribute to Raised mammographic density. *Breast Cancer Res.* **18**, 5 (2016). 26747277 10.1186/s13058-015-0664-2 PMC4706673

[11] Boyd, N. F. et al. Breast tissue composition and susceptibility to breast cancer. *J. Natl. Cancer Inst.* **102**, 1224–1237 (2010). 20616353 10.1093/jnci/djq239 PMC2923218

[12] Northey, J. J. et al. Stiff stroma increases breast cancer risk by inducing the oncogene ZNF217. *J Clin. Invest. ***130**, 5721–5737 (2020). 10.1172/JCI129249 PMC7598051 32721948

[13] Chaudhuri, O. et al. Extracellular matrix stiffness and composition jointly regulate the induction of malignant phenotypes in mammary epithelium. *Nat. Mater.* **13**, 970 (2014). 24930031 10.1038/nmat4009

[14] Paszek, M. J. et al. Tensional homeostasis and the malignant phenotype. *Cancer Cell.* **8**, 241–254 (2005). 16169468 10.1016/j.ccr.2005.08.010

[15] Aisenbrey, E. A. & Murphy, W. L. Synthetic alternatives to matrigel. *Nat. Rev. Mater.* **5**, 539–551 (2020). 32953138 10.1038/s41578-020-0199-8 PMC7500703

[16] Reed, J., Walczak, W. J., Petzold, O. N. & Gimzewski, J. K. In situ mechanical interferometry of matrigel films. *Langmuir* **25**, 36–39 (2009). 19049394 10.1021/la8033098 PMC3437936

[17] Fialka, I. et al. The estrogen-dependent c-JunER protein causes a reversible loss of mammary epithelial cell Polarity involving a destabilization of adherens junctions. *J. Cell. Biol.* **132**, 1115–1132 (1996). 8601589 10.1083/jcb.132.6.1115 PMC2120757

[18] Wang, P. et al. Vinculins interaction with Talin is essential for mammary epithelial differentiation. *Sci. Rep.* **9**, 18400 (2019). 31804547 10.1038/s41598-019-54784-w PMC6895056

[19] Wysolmerski, J. J. Parathyroid hormone-related protein: an update. *J. Clin. Endocrinol. Metab.* **97**, 2947–2956 (2012). 22745236 10.1210/jc.2012-2142 PMC3431578

[20] Borowicz, S. et al. The soft agar colony formation assay. *J Vis. Exp. ***92**, e51998 (2014). 10.3791/51998 PMC4353381 25408172

[21] Sharma, A., Singh, K. & Almasan, A. Histone H2AX phosphorylation: a marker for DNA damage. *Methods Mol. Biol.* **920**, 613–626 (2012). 22941631 10.1007/978-1-61779-998-3_40

[22] Park, J. S. et al. Mechanical regulation of Glycolysis via cytoskeleton architecture. *Nature* **578**, 621–626 (2020). 32051585 10.1038/s41586-020-1998-1 PMC7210009

[23] Romani, P. et al. Extracellular matrix mechanical cues regulate lipid metabolism through Lipin-1 and SREBP. *Nat. Cell Biol.* **21**, 338–347 (2019). 30718857 10.1038/s41556-018-0270-5

[24] Voulgaridou, G. P., Anestopoulos, I., Franco, R., Panayiotidis, M. I. & Pappa A. DNA damage induced by endogenous aldehydes: current state of knowledge. *Mutat. Res.* **711**, 13–27 (2011). 21419140 10.1016/j.mrfmmm.2011.03.006

[25] Ginestier, C. et al. ALDH1 is a marker of normal and malignant human mammary stem cells and a predictor of poor clinical outcome. *Cell. Stem Cell.* **1**, 555–567 (2007). 18371393 10.1016/j.stem.2007.08.014 PMC2423808

[26] Michorowska, S. et al. Detection of ALDH3B2 in human placenta. *Int. J. Mol. Sci.* **20**, 6292 (2019). 31847104 10.3390/ijms20246292 PMC6941052

[27] Zanconato, F., Cordenonsi, M. & Piccolo, S. YAP/TAZ at the roots of Cancer. *Cancer Cell.* **29**, 783–803 (2016). 27300434 10.1016/j.ccell.2016.05.005 PMC6186419

[28] Zhao, B. et al. Inactivation of YAP oncoprotein by the Hippo pathway is involved in cell contact Inhibition and tissue growth control. *Genes Dev.* **21**, 2747–2761 (2007). 17974916 10.1101/gad.1602907 PMC2045129

[29] Liu-Chittenden, Y. et al. Genetic and Pharmacological disruption of the TEAD-YAP complex suppresses the oncogenic activity of YAP. *Genes Dev.* **26**, 1300–1305 (2012). 22677547 10.1101/gad.192856.112 PMC3387657

[30] Chin, L., Xia, Y., Discher, D. E. & Janmey, P. A. Mechanotransduction in cancer. *Curr. Opin. Chem. Eng.* **11**, 77–84 (2016). 28344926 10.1016/j.coche.2016.01.011 PMC5362117

[31] Pors, K. & Moreb, J. S. Aldehyde dehydrogenases in cancer: an opportunity for biomarker and drug development? *Drug Discovery Today*. **19**, 1953–1963 (2014). 25256776 10.1016/j.drudis.2014.09.009

[32] Croker, A. K. et al. Differential functional roles of ALDH1A1 and ALDH1A3 in mediating metastatic behavior and therapy resistance of human breast Cancer cells. *Int. J. Mol. Sci.* **18**, 2039 (2017). 28937653 10.3390/ijms18102039 PMC5666721

[33] Boyd, N. F. et al. Plasma lipids, lipoproteins, and mammographic densities. *Cancer Epidemiol. Biomarkers Prev.* **4**, 727–733 (1995). 8672989

[34] Boyd, N. F. & McGuire, V. Evidence of lipid peroxidation in premenopausal women with mammographic dysplasia. *Cancer Lett.* **50**, 31–37 (1990). 2322925 10.1016/0304-3835(90)90175-w

[35] DeFilippis, R. A. et al. Stress signaling from human mammary epithelial cells contributes to phenotypes of mammographic density. *Cancer Res.* **74**, 5032–5044 (2014). 25172842 10.1158/0008-5472.CAN-13-3390 PMC4335659

[36] Aragona, M. et al. A mechanical checkpoint controls multicellular growth through YAP/TAZ regulation by actin-processing factors. *Cell* **154**, 1047–1059 (2013). 23954413 10.1016/j.cell.2013.07.042

[37] Shiu, J. Y., Aires, L., Lin, Z. & Vogel, V. Nanopillar force measurements reveal actin-cap-mediated YAP mechanotransduction. *Nat. Cell Biol.* **20**, 262–271 (2018). 29403039 10.1038/s41556-017-0030-y

[38] Elosegui-Artola, A. et al. Force triggers YAP nuclear entry by regulating transport across nuclear pores. *Cell.* **171**, 1397–1410e1314 (2017). 29107331 10.1016/j.cell.2017.10.008

[39] Lee, J. Y. et al. Identification of cell context-dependent YAP-associated proteins reveals β1 and β4 integrin mediate YAP translocation independently of cell spreading. *Sci. Rep.* **9**, 17188 (2019). 31748579 10.1038/s41598-019-53659-4 PMC6868278

[40] Lee, J. Y. et al. YAP-independent mechanotransduction drives breast cancer progression. *Nat. Commun.* **10**, 1848 (2019). 31015465 10.1038/s41467-019-09755-0 PMC6478686

[41] Panciera, T. et al. Reprogramming normal cells into tumour precursors requires ECM stiffness and oncogene-mediated changes of cell mechanical properties. *Nature Materials. ***19**, 797–806 (2020). 10.1038/s41563-020-0615-x PMC7316573 32066931

[42] Burridge, K., Monaghan-Benson, E. & Graham, D. M. Mechanotransduction: from the cell surface to the nucleus via RhoA. *Philos. Trans. R Soc. Lond. B Biol. Sci.* **374**, 20180229 (2019). 31431179 10.1098/rstb.2018.0229 PMC6627015

[43] Haikala, H. M., Marques, E., Turunen, M. & Klefstrom, J. Myc requires rhoa/srf to reprogram glutamine metabolism. *Small GTPases*. **9**, 274–282 (2018). 27532209 10.1080/21541248.2016.1224287 PMC5927483

[44] Bertolio, R. et al. Sterol regulatory element binding protein 1 couples mechanical cues and lipid metabolism. *Nat. Commun.* **10**, 1326 (2019). 30902980 10.1038/s41467-019-09152-7 PMC6430766

[45] Bagci, H. et al. Mapping the proximity interaction network of the Rho-family GTPases reveals signalling pathways and regulatory mechanisms. *Nat. Cell. Biol.* **22**, 120–134 (2020). 31871319 10.1038/s41556-019-0438-7

[46] Zhao, B. et al. TEAD mediates YAP-dependent gene induction and growth control. *Genes Dev.* **22**, 1962–1971 (2008). 18579750 10.1101/gad.1664408 PMC2492741

[47] Guo, W. et al. Slug and Sox9 cooperatively determine the mammary stem cell state. *Cell* **148**, 1015–1028 (2012). 22385965 10.1016/j.cell.2012.02.008 PMC3305806

[48] Bolger, A. M., Lohse, M. & Usadel, B. Trimmomatic: a flexible trimmer for illumina sequence data. *Bioinformatics* **30**, 2114–2120 (2014). 24695404 10.1093/bioinformatics/btu170 PMC4103590

[49] Dobin, A. et al. STAR: ultrafast universal RNA-seq aligner. *Bioinformatics* **29**, 15–21 (2013). 23104886 10.1093/bioinformatics/bts635 PMC3530905

[50] Anders, S., Pyl, P. T. & Huber, W. HTSeq–a Python framework to work with high-throughput sequencing data. *Bioinformatics* **31**, 166–169 (2015). 25260700 10.1093/bioinformatics/btu638 PMC4287950

[51] Love, M. I., Huber, W. & Anders, S. Moderated Estimation of fold change and dispersion for RNA-seq data with DESeq2. *Genome Biol.* **15**, 550 (2014). 25516281 10.1186/s13059-014-0550-8 PMC4302049

[52] Alexa, A., Rahnenfuhrer, J. & Lengauer, T. Improved scoring of functional groups from gene expression data by decorrelating GO graph structure. *Bioinformatics* **22**, 1600–1607 (2006). 16606683 10.1093/bioinformatics/btl140

[53] Benjamini, Y. & Hochberg, Y. Controlling the false discovery rate: A practical and powerful approach to multiple testing. *J. Royal Stat. Soc. Ser. B (Methodological)*. **57**, 289–300 (1995).

[54] Yu, G., Wang, L. G., Han, Y. & He, Q. Y. ClusterProfiler: an R package for comparing biological themes among gene clusters. *OMICS* **16**, 284–287 (2012). 22455463 10.1089/omi.2011.0118 PMC3339379

[55] Wang, J. Z., Du, Z., Payattakool, R., Yu, P. S. & Chen, C. F. A new method to measure the semantic similarity of GO terms. *Bioinformatics* **23**, 1274–1281 (2007). 17344234 10.1093/bioinformatics/btm087

[56] Herrera, J. A. et al. Laser capture microdissection coupled mass spectrometry (LCM-MS) for spatially resolved analysis of formalin-fixed and stained human lung tissues. *Clin. Proteomics*. **17**, 24 (2020). 32565759 10.1186/s12014-020-09287-6 PMC7302139

[57] Tyanova, S., Temu, T. & Cox, J. The MaxQuant computational platform for mass spectrometry-based shotgun proteomics. *Nat. Protoc.* **11**, 2301–2319 (2016). 27809316 10.1038/nprot.2016.136

[58] Goeminne, L. J., Gevaert, K. & Clement, L. Peptide-level robust ridge regression improves estimation, sensitivity, and specificity in Data-dependent quantitative Label-free shotgun proteomics. *Mol. Cell. Proteom.* **15**, 657–668 (2016). 10.1074/mcp.M115.055897 PMC4739679 26566788

[59] Goeminne, L. J. E., Sticker, A., Martens, L., Gevaert, K. & Clement, L. MSqRob takes the missing hurdle: uniting Intensity- and Count-Based proteomics. *Anal. Chem.* **92**, 6278–6287 (2020). 32227882 10.1021/acs.analchem.9b04375

[60] Hynes, R. O. & Naba, A. Overview of the matrisome–an inventory of extracellular matrix constituents and functions. *Cold Spring Harb Perspect. Biol.* **4**, a004903 (2012). 21937732 10.1101/cshperspect.a004903 PMC3249625

[61] Naba, A., Hoersch, S. & Hynes, R. O. Towards definition of an ECM parts list: an advance on GO categories. *Matrix Biol.* **31**, 371–372 (2012). 23199376 10.1016/j.matbio.2012.11.008 PMC4116136

[62] Sievers, F. et al. Fast, scalable generation of high-quality protein multiple sequence alignments using clustal Omega. *Mol. Syst. Biol.* **7**, 539 (2011). 21988835 10.1038/msb.2011.75 PMC3261699

[63] Mi, H. et al. PANTHER version 16: a revised family classification, tree-based classification tool, enhancer regions and extensive API. *Nucleic Acids Res.* **49**, D394–D403 (2021). 33290554 10.1093/nar/gkaa1106 PMC7778891

[64] Bindea, G. et al. ClueGO: a cytoscape plug-in to Decipher functionally grouped gene ontology and pathway annotation networks. *Bioinformatics* **25**, 1091–1093 (2009). 19237447 10.1093/bioinformatics/btp101 PMC2666812

[65] Shannon, P. et al. Cytoscape: a software environment for integrated models of biomolecular interaction networks. *Genome Res.* **13**, 2498–2504 (2003). 14597658 10.1101/gr.1239303 PMC403769

[66] Bankhead, P. et al. QuPath: open source software for digital pathology image analysis. *Sci. Rep.* **7**, 16878 (2017). 29203879 10.1038/s41598-017-17204-5 PMC5715110

[67] Schindelin, J. et al. Fiji: an open-source platform for biological-image analysis. *Nat. Methods*. **9**, 676–682 (2012). 22743772 10.1038/nmeth.2019 PMC3855844

