# Supplementary material for: Matrix stiffness drives alterations in aldehyde metabolism, inducing DNA damage and transformation
Source: Sci Rep. 2025 Aug 9;15:29127. doi: 10.1038/s41598-025-12880-0 (PMC12334586; doi:10.1038/s41598-025-12880-0)
Supplement: Supplementary file 1 — Supplementary Material 1 [file 41598_2025_12880_MOESM1_ESM.pdf]

Supplementary data

Matrix stiffness drives alterations in aldehyde metabolism, inducing DNA damage and transformation

Matthew Jones[1,2,*], Hannah Percival[2*], Alis Hales[1,2*], Amber Wood[2*], Heyuan Sun[1,2], Fabianna Tennant[1,2], Eleanor Broadberry[2], Eldhose Skaria[1], Harry Barnes[2], Egor Zindy[1,4], Craig Lawless[1], Charles Streuli[1], Joe Swift[1,3], Keith Brennan[2#], and Andrew P. Gilmore[2#*]

* these authors contributed equally to the work

# Joint senior authors

1. Wellcome Trust Centre for Cell-Matrix Research, Faculty of Biology, Medicine and Health University of Manchester, Manchester, UK.
2. Division of Cancer Sciences, Faculty of Biology, Medicine and Health, University of Manchester, Manchester, UK.
3. Division of Cell Matrix Biology and Regenerative Medicine, Faculty of Biology, Medicine and Health, University of Manchester, Manchester, UK.
4. Present address: Center for Microscopy and Molecular Imaging, Université libre de Bruxelles, Gosselies B-6041, Belgium

Supplementary Figure legends

Figure S1.

A. Log_2 fold-change in gene expression of *Pthrp*, normalised to *Gapdh*, for EpH4 acini grown for 10 days in Matrigel-Alginate hydrogels with 0 mM, 2,4 mM and 24 mM $CaSO_4$, as determined by RT-qPCR. Mean ± SD, n = 3 per condition, across independent experiments (represented by data points). Two-way ANOVA with Tukey's post-hoc test.

B. Log_2 fold-change in gene expression of mammary cell lineage markers as determined by RT-qPCR in EpH4 cells grown for 10 days in Matrigel-Alginate hydrogels, with 0 mM or 2.4 mM $CaSO_4$. Mean ± SD, n = 3 for each condition across independent experiments (represented by data points). Two-way ANOVA with Tukey's post-hoc test.

Figure S2

A. Hierarchical clustering heatmap representing significantly differentially expressed genes in EpH4 cells cultured in 2D gels of different stiffnesses, vs 3D gels of different stiffnesses, as determined by RNAseq. Three independent biological replicates of both soft and stiff 2D and 3D cultures are shown

B. MA plot generated from RNAseq data, showing genes that are significantly upregulated (blue) and downregulated (red) in EpH4 cells grown in the stiff condition, relative to soft in 2D and 3D. Data shown are the mean from the 3 biological replicates in (a) for both 2D and 3D

C. Top panel shows Log_2 fold-change in expression of genes associated with milk production and PTHLH in EpH4 cells grown in the stiff condition relative to soft, as determined by RNAseq. Lower panel shows Log_2 fold-change in expression of the same genes comparing soft 2D hydrogel with soft 3D. Error bars represent SE (n = 3), statistical significance was determined using DESeq2.

D. Alignment of the murine protein sequences of Aldh3b1 and Aldh3b2 using the Clustal Omega server. Sequences highlighted in yellow represent peptides for each protein identified in the mass spectrometry analysis. Residues in purple form part of the active site and are essential for catalysis; residues in blue are required for Rossman fold formation and co-factor binding; residues in grey ensure correct geometry of the active site (Michorowska et al., 2019)

Figure S3

A. Log_2 fold-change in expression of genes involved in the Base Excision Repair pathway in EpH4 cells grown in the stiff condition relative to soft, as determined by RNAseq. Error bars represent SE (n = 3). Statistical significance was determined using DESeq2.

B. Log_2 fold-change in expression of genes involved in the Nucleotide Excision Repair pathway in EpH4 cells grown in the stiff condition relative to soft, as determined by RNAseq. Error bars represent SE (n = 3). Statistical significance was determined using DESeq2.

C. Log_2 fold-change in expression of genes involved in the Homologous Recombination DNA repair in EpH4 cells grown in the stiff condition relative to soft, as determined by RNAseq. Error bars represent SE (n = 3). Statistical significance was determined using DESeq2.

D. Log_2 fold-change in expression of genes involved in the Mismatch Repair pathway in EpH4 cells grown in the stiff condition relative to soft, as determined by RNAseq. Error bars represent SE (n = 3). Statistical significance was determined using DESeq2.

E. Log_2 fold-change in expression of genes involved in Non-Homologous End-Joining DNA repair in EpH4 cells grown in the stiff condition relative to soft, as determined by RNAseq. Error bars represent SE (n = 3). Statistical significance was determined using DESeq2.

Figure S4.

A. Bar chart showing all GO terms within each functional group in the ClueGO analysis in Figure 3D. Single (*) or double (**) asterisks indicate significantly enriched GO terms at the $p < 0.05$ and $p < 0.01$ statistical levels, respectively. The numbers annotated on the bars are the number of genes associated with that GO term. The percentage genes per term (x-axis) refers to the percentage of genes that were mapped to that GO term in the GO analysis out of all genes in the input dataset that are associated with that GO term. The number of associated genes that are mapped to the GO term in the analysis depends on the network specificity settings which are set before the network is generated. In this case, a network specificity of "medium" has been selected, where mapped identifiers represent a minimum of 4% of total associated genes with the term. Figure generated using the ClueGO plugin within Cytoscape (v.2.5.10), accessed February 2023.

B. Bar chart showing GO terms within each functional group in the ClueGO analysis in Figure 4C. Single (*) or double (**) asterisks indicate significantly enriched GO terms at the $p < 0.05$ and $p < 0.01$ statistical levels, respectively. The numbers annotated on the bars are the number of proteins associated with that GO term. The percentage genes per term (x-axis)

refers to the percentage of proteins that were mapped to that GO term in the GO analysis out of all proteins in the input dataset that are associated with that GO term. Figures were generated using the ClueGO plugin within Cytoscape (v.2.5.10), accessed February 2023.

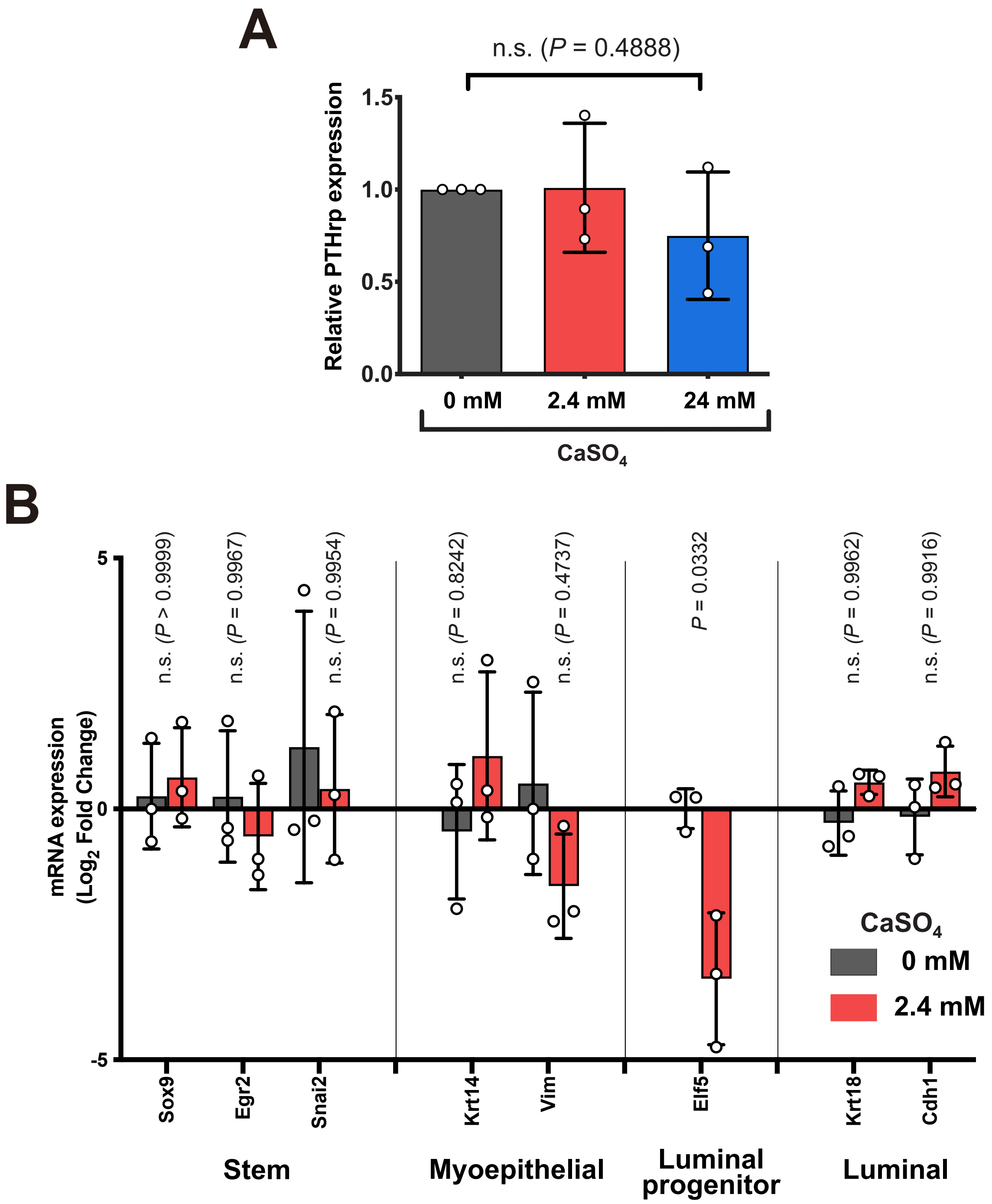
A
n.s. (P = 0.4888)
Relative PTHrp expression
1.5
1.0
0.5
0.0
0 mM
2.4 mM
24 mM
CaSO4
B
mRNA expression (Log2 Fold Change)
5
0
-5
n.s. (P > 0.9999)
n.s. (P = 0.9967)
n.s. (P = 0.9954)
n.s. (P = 0.8242)
n.s. (P = 0.4737)
P = 0.0332
n.s. (P = 0.9962)
n.s. (P = 0.9916)
Sox9
Egr2
Snai2
Krt14
Vim
Elf5
Krt18
Cdh1
Stem
Myoepithelial
Luminal progenitor
Luminal
CaSO4
0 mM
2.4 mM

Figure S1

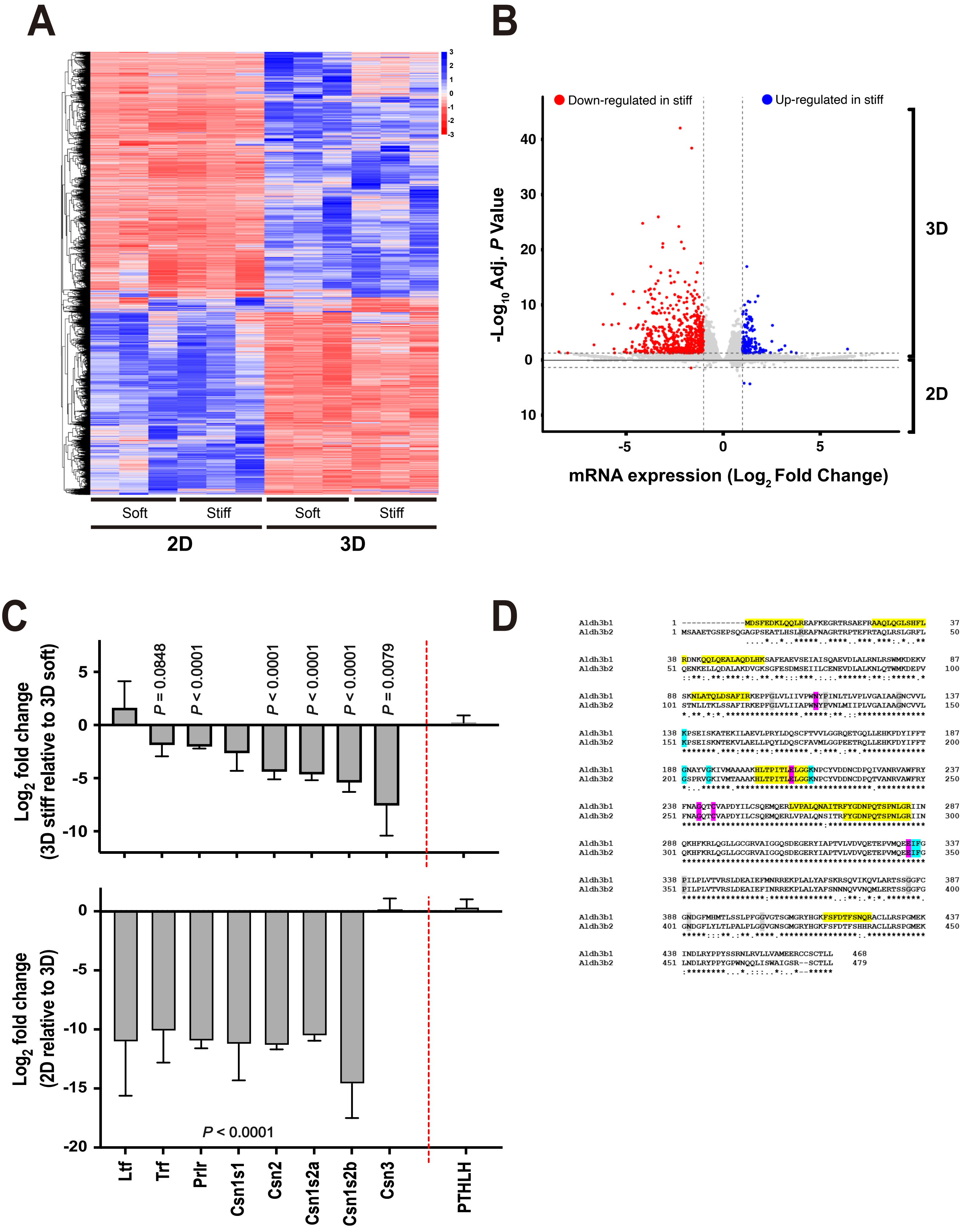
A
B
C
D
3
2
1
0
-1
-2
-3
Soft
Stiff
Soft
Stiff
2D
3D
Down-regulated in stiff
Up-regulated in stiff
-Log10 Adj. P Value
40
30
20
10
0
10
-5
0
5
mRNA expression (Log2 Fold Change)
3D
2D
Log2 fold change (3D stiff relative to 3D soft)
P = 0.0848
P < 0.0001
P < 0.0001
P < 0.0001
P < 0.0001
P = 0.0079
5
0
-5
-10
Log2 fold change (2D relative to 3D)
0
-5
-10
-15
-20
P < 0.0001
Ltf
Trf
Prlr
Csn1s1
Csn2
Csn1s2a
Csn1s2b
Csn3
PTHLH
Aldh3b1 1 -------------MDSFEDKLQQLREAFKEGRTRSAEFRAAQLQGLSHFL 37
Aldh3b2 1 MSAAETGSEPSQGAGPSEATLHSLREAFNAGRTRPTEFRTAQLRSLGRFL 50
Aldh3b1 38 RDNKQQLQEALAQDLHKSAFEAEVSEIAISQAEVDLALRNLRSWMKDEKV 87
Aldh3b2 51 QENKELLQDALAKDVGKSGFESDMSEIILCENEVDLALKNLQTWMKDEPV 100
Aldh3b1 88 SKNLATQLDSAFIRKEPFGLVLIIVPWNYPINLTLVPLVGAIAAGNCVVL 137
Aldh3b2 101 STNLLTKLSSAFIRKEPFGLVLIIAPWNYPVNLMIIPLVGAIAAGNCVVL 150
Aldh3b1 138 KPSEISKATEKILAEVLPRYLDQSCFTVVLGGRQETGQLLEHKFDYIFFT 187
Aldh3b2 151 KPSEISKNTEKVLAELLPQYLDQSCFAVMLGGPEETRQLLEHKFDYIFFT 200
Aldh3b1 188 GNAYVGKIVMAAAAKHLTPITLELGGKNPCYVDDNCDPQIVANRVAWFRY 237
Aldh3b2 201 GSPRVGKIVMTAAAKHLTPITLELGGKNPCYVDDNCDPQTVANRVAWFRY 250
Aldh3b1 238 FNAGQTCVAPDYILCSQEMQERLVPALQNAITRFYGDNPQTSPNLGRIIN 287
Aldh3b2 251 FNAGQTCVAPDYILCSQEMQERLVPALQNSITRFYGDNPQTSPNLGRIIN 300
Aldh3b1 288 QKHFKRLQGLLGCGRVAIGGQSDEGERYIAPTVLVDVQETEPVMQEEIFG 337
Aldh3b2 301 QKHFKRLQGLLGCGRVAIGGQSDEGERYIAPTVLVDVQETEPVMQEEIFG 350
Aldh3b1 338 PILPLVTVRSLDEAIEFMNRREKPLALYAFSKRSQVIKQVLARTSSGGFC 387
Aldh3b2 351 PILPLVTVRSLDEAIEFINRREKPLALYAFSNNNQVVNQMLERTSSGGFG 400
Aldh3b1 388 GNDGFMHMTLSSLPFGGVGTSGMGRYHGKFSFDTFSNQRACLLRSPGMEK 437
Aldh3b2 401 GNDGFLYLTLPALPLGGVGNSGMGRYHGKFSFDTFSHHRACLLRSPGMEK 450
Aldh3b1 438 INDLRYPPYSSRNLRVLLVAMEERCCSCTLL 468
Aldh3b2 451 LNDLRYPPYGPWNQQLISWAIGSR--SCTLL 479

Figure S2

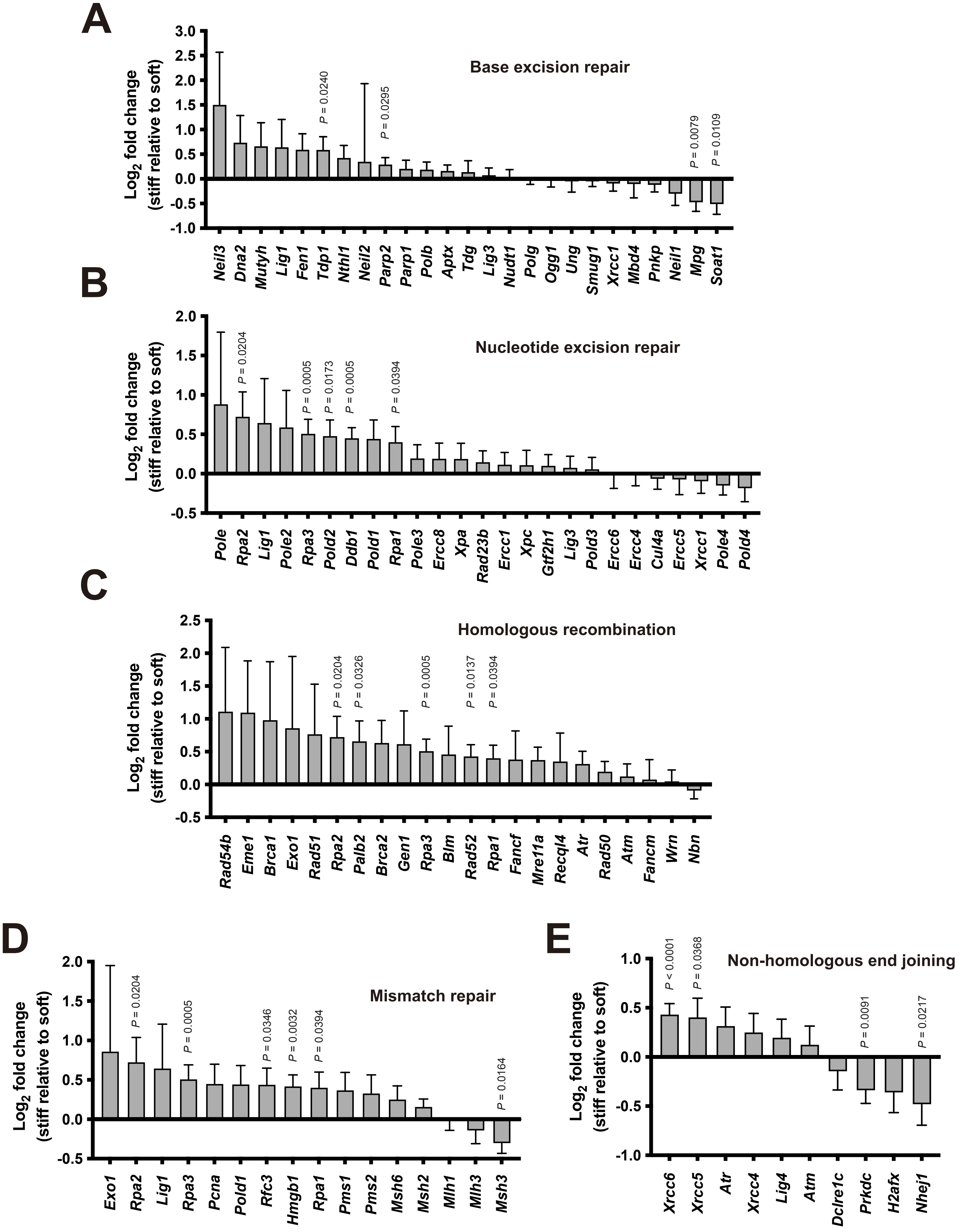
A
Base excision repair
Log2 fold change (stiff relative to soft)
3.0
2.5
2.0
1.5
1.0
0.5
0.0
-0.5
-1.0
P = 0.0240
P = 0.0295
P = 0.0079
P = 0.0109
Neil3 Dna2 Mutyh Lig1 Fen1 Tdp1 Nthl1 Neil2 Parp2 Parp1 Polb Aptx Tdg Lig3 Nudt1 Polg Ogg1 Ung Smug1 Xrcc1 Mbd4 Pnkp Neil1 Mpg Soat1
B
Nucleotide excision repair
Log2 fold change (stiff relative to soft)
2.0
1.5
1.0
0.5
0.0
-0.5
P = 0.0204
P = 0.0005
P = 0.0173
P = 0.0005
P = 0.0394
Pole Rpa2 Lig1 Pole2 Rpa3 Pold2 Ddb1 Pold1 Rpa1 Pole3 Ercc8 Xpa Rad23b Ercc1 Xpc Gtf2h1 Lig3 Pold3 Ercc6 Ercc4 Cul4a Ercc5 Xrcc1 Pole4 Pold4
C
Homologous recombination
Log2 fold change (stiff relative to soft)
2.5
2.0
1.5
1.0
0.5
0.0
-0.5
P = 0.0204
P = 0.0326
P = 0.0005
P = 0.0137
P = 0.0394
Rad54b Eme1 Brca1 Exo1 Rad51 Rpa2 Palb2 Brca2 Gen1 Rpa3 Blm Rad52 Rpa1 Fancf Mre11a Recql4 Atr Rad50 Atm Fancm Wrn Nbn
D
Mismatch repair
Log2 fold change (stiff relative to soft)
2.0
1.5
1.0
0.5
0.0
-0.5
P = 0.0204
P = 0.0005
P = 0.0346
P = 0.0032
P = 0.0394
P = 0.0164
Exo1 Rpa2 Lig1 Rpa3 Pcna Pold1 Rfc3 Hmgb1 Rpa1 Pms1 Pms2 Msh6 Msh2 Mlh1 Mlh3 Msh3
E
Non-homologous end joining
Log2 fold change (stiff relative to soft)
1.0
0.5
0.0
-0.5
-1.0
P < 0.0001
P = 0.0368
P = 0.0091
P = 0.0217
Xrcc6 Xrcc5 Atr Xrcc4 Lig4 Atm Dclre1c Prkdc H2afx Nhej1

Figure S3

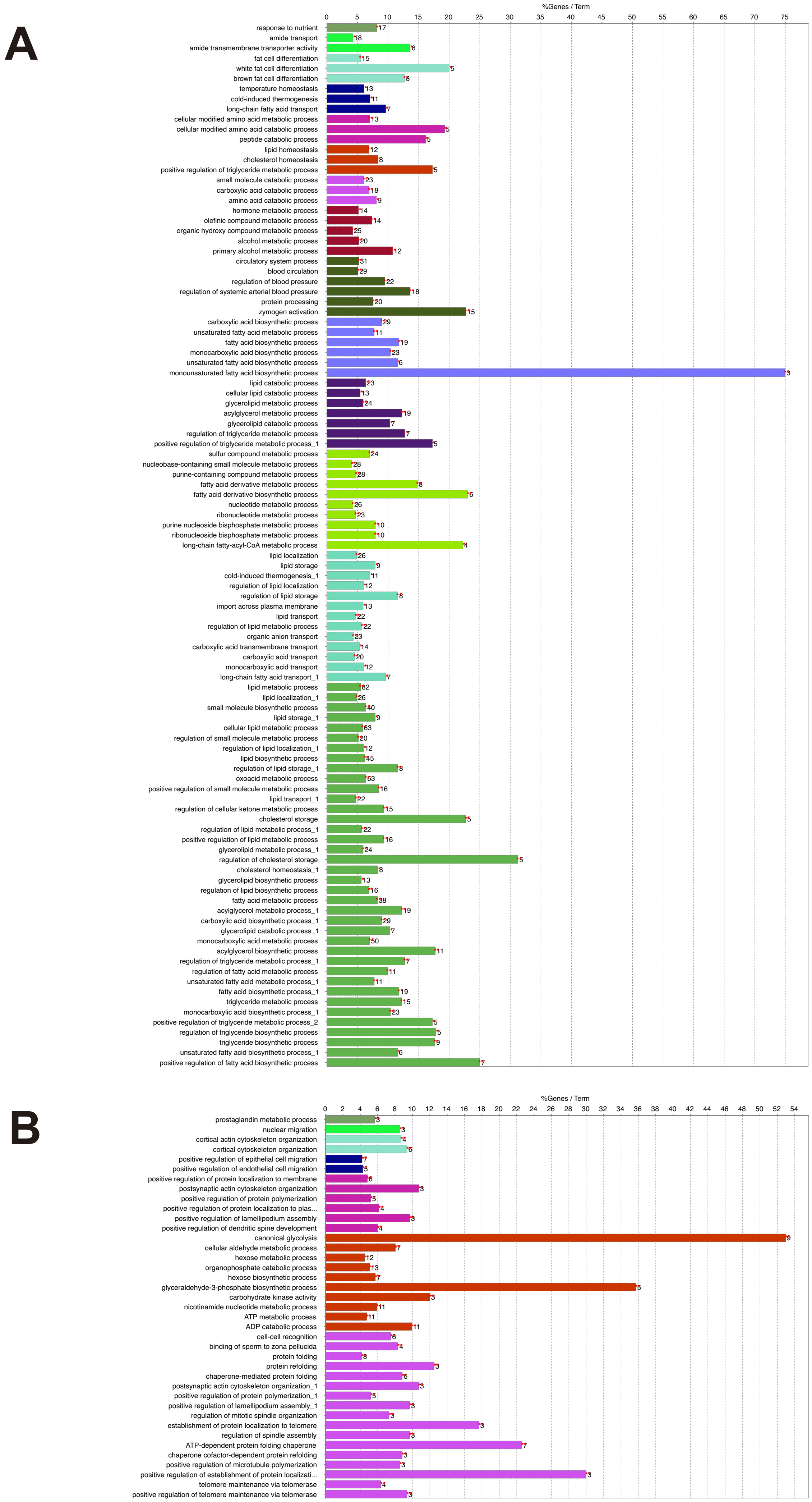
A
%Genes / Term
response to nutrient
amide transport
amide transmembrane transporter activity
fat cell differentiation
white fat cell differentiation
brown fat cell differentiation
temperature homeostasis
cold-induced thermogenesis
long-chain fatty acid transport
cellular modified amino acid metabolic process
cellular modified amino acid catabolic process
peptide catabolic process
lipid homeostasis
cholesterol homeostasis
positive regulation of triglyceride metabolic process
small molecule catabolic process
carboxylic acid catabolic process
amino acid catabolic process
hormone metabolic process
olefinic compound metabolic process
organic hydroxy compound metabolic process
alcohol metabolic process
primary alcohol metabolic process
circulatory system process
blood circulation
regulation of blood pressure
regulation of systemic arterial blood pressure
protein processing
zymogen activation
carboxylic acid biosynthetic process
unsaturated fatty acid metabolic process
fatty acid biosynthetic process
monocarboxylic acid biosynthetic process
unsaturated fatty acid biosynthetic process
monounsaturated fatty acid biosynthetic process
lipid catabolic process
cellular lipid catabolic process
glycerolipid metabolic process
acylglycerol metabolic process
glycerolipid catabolic process
regulation of triglyceride metabolic process
positive regulation of triglyceride metabolic process_1
sulfur compound metabolic process
nucleobase-containing small molecule metabolic process
purine-containing compound metabolic process
fatty acid derivative metabolic process
fatty acid derivative biosynthetic process
nucleotide metabolic process
ribonucleotide metabolic process
purine nucleoside bisphosphate metabolic process
ribonucleoside bisphosphate metabolic process
long-chain fatty-acyl-CoA metabolic process
lipid localization
lipid storage
cold-induced thermogenesis_1
regulation of lipid localization
regulation of lipid storage
import across plasma membrane
lipid transport
regulation of lipid metabolic process
organic anion transport
carboxylic acid transmembrane transport
carboxylic acid transport
monocarboxylic acid transport
long-chain fatty acid transport_1
lipid metabolic process
lipid localization_1
small molecule biosynthetic process
lipid storage_1
cellular lipid metabolic process
regulation of small molecule metabolic process
regulation of lipid localization_1
lipid biosynthetic process
regulation of lipid storage_1
oxoacid metabolic process
positive regulation of small molecule metabolic process
lipid transport_1
regulation of cellular ketone metabolic process
cholesterol storage
regulation of lipid metabolic process_1
positive regulation of lipid metabolic process
glycerolipid metabolic process_1
regulation of cholesterol storage
cholesterol homeostasis_1
glycerolipid biosynthetic process
regulation of lipid biosynthetic process
fatty acid metabolic process
acylglycerol metabolic process_1
carboxylic acid biosynthetic process_1
glycerolipid catabolic process_1
monocarboxylic acid metabolic process
acylglycerol biosynthetic process
regulation of triglyceride metabolic process_1
regulation of fatty acid metabolic process
unsaturated fatty acid metabolic process_1
fatty acid biosynthetic process_1
triglyceride metabolic process
monocarboxylic acid biosynthetic process_1
positive regulation of triglyceride metabolic process_2
regulation of triglyceride biosynthetic process
triglyceride biosynthetic process
unsaturated fatty acid biosynthetic process_1
positive regulation of fatty acid biosynthetic process
B
%Genes / Term
prostaglandin metabolic process
nuclear migration
cortical actin cytoskeleton organization
cortical cytoskeleton organization
positive regulation of epithelial cell migration
positive regulation of endothelial cell migration
positive regulation of protein localization to membrane
postsynaptic actin cytoskeleton organization
positive regulation of protein polymerization
positive regulation of protein localization to plas...
positive regulation of lamellipodium assembly
positive regulation of dendritic spine development
canonical glycolysis
cellular aldehyde metabolic process
hexose metabolic process
organophosphate catabolic process
hexose biosynthetic process
glyceraldehyde-3-phosphate biosynthetic process
carbohydrate kinase activity
nicotinamide nucleotide metabolic process
ATP metabolic process
ADP catabolic process
cell-cell recognition
binding of sperm to zona pellucida
protein folding
protein refolding
chaperone-mediated protein folding
postsynaptic actin cytoskeleton organization_1
positive regulation of protein polymerization_1
positive regulation of lamellipodium assembly_1
regulation of mitotic spindle organization
establishment of protein localization to telomere
regulation of spindle assembly
ATP-dependent protein folding chaperone
chaperone cofactor-dependent protein refolding
positive regulation of microtubule polymerization
positive regulation of establishment of protein localizati...
telomere maintenance via telomerase
positive regulation of telomere maintenance via telomerase

Figure S4
